# Immunotherapy for TKI-resistant, EGFR L858R-mutated non-small cell lung cancer: a systematic review and meta-analysis of randomized and single-arm studies

**DOI:** 10.3389/fimmu.2026.1787310

**Published:** 2026-04-10

**Authors:** Peipei Zhang, Weixing Zhao, Jiayun Ma, Yuan Li, Huiyuan Peng, Jun Jiang

**Affiliations:** Department of Medical Oncology, Qinghai University Affiliated Hospital, Qinghai, China

**Keywords:** EGFR L858R mutation, immune checkpoint inhibitors, non-small cell lung cancer, single-arm meta-analysis, tyrosine kinase inhibitor resistance

## Abstract

**Objective:**

The application of immune checkpoint inhibitors (ICIs) in epidermal growth factor receptor (EGFR)-mutated non-small cell lung cancer (NSCLC) following tyrosine kinase inhibitor (TKI) resistance remains controversial. Prior studies often obscured immunobiological heterogeneity by analyzing exon 19 deletions and L858R mutations in aggregate. This study conducts the first meta-analysis specifically focusing on the EGFR L858R subtype to systematically evaluate ICI efficacy (monotherapy vs. combinations) in the post-TKI setting.

**Methods:**

We systematically searched PubMed, EMBASE, Cochrane, and Web of Science (up to January 1, 2026) for studies on ICI-treated EGFR-mutated NSCLC. Prospective or retrospective studies explicitly reporting L858R data were selected. Primary endpoints included progression-free survival (PFS) and objective response rate (ORR); the secondary endpoint was overall survival (OS). Given that our meta-analysis includes both randomized controlled trials and single-arm studies, we explicitly stratified analyses by study design. RCTs provide comparative estimates, whereas single-arm studies are descriptive and exploratory; pooled results from single-arm studies should not be interpreted as definitive evidence of efficacy. Pooled hazard ratios (HRs) were calculated using a random-effects model, stratified by treatment modality.

**Results:**

Seventeen studies involving 1, 154 patients were included. Compared with chemotherapy, ICI-based treatments significantly improved PFS [HR = 0.63, 95% confidence intervals (CI):0.44-0.90, P = 0.01]. Subgroup analysis revealed significant disparities: ICI monotherapy failed to confer a PFS benefit (HR = 1.68, 95% CI:0.94-2.99, P = 0.08), whereas combination therapies were effective. Exploratory analyses from non-comparative single-arm studies supported these trends, showing higher ORR and PFS for combination regimens than for monotherapy. Notably, the “ICI plus anti-angiogenic agents plus chemotherapy” regimen exhibited the optimal PFS benefit (HR = 0.50, P<0.01), outperforming “ICI plus chemotherapy” (HR = 0.57, P = 0.02). Combination therapy also yielded superior ORR. However, no statistically significant OS advantage was observed for ICI treatment versus chemotherapy (HR = 0.97, P = 0.83).

**Conclusion:**

For EGFR-TKI-resistant, L858R-mutated NSCLC, ICI monotherapy appears ineffective. Conversely, ICI-based combinations—particularly the four-drug regimen incorporating anti-angiogenic agents—significantly delay disease progression and improve response rates. Future research should prioritize prospective studies and novel agents, such as antibody-drug conjugates, to improve long-term survival.

**Systematic Review Registration:**

https://www.crd.york.ac.uk/PROSPERO/view/, identifier CRD420261278330.

## 1 Introduction

Lung cancer continues to present a formidable challenge to global public health. According to the latest statistics from the International Agency for Research on Cancer, it remains the leading cause of cancer-related mortality worldwide. With over two million new cases diagnosed annually, non-small cell lung cancer (NSCLC) accounts for approximately 80% to 85% of the total disease burden, contributing significantly to the persistently high rates of both incidence and mortality (1, 2). In patients with NSCLC, epidermal growth factor receptor (EGFR) gene mutations represent one of the most frequent driver alterations, particularly among Asian non-smoking patients with adenocarcinoma, where the prevalence approaches 50% (3). Within this mutational landscape, the L858R point mutation in exon 21 and the exon 19 deletion (19-del) are the two most canonical activating mutations. Collectively, these subtypes account for the vast majority of all EGFR mutations, with L858R comprising approximately 40% to 45% of cases (4).

The development of EGFR tyrosine kinase inhibitors (EGFR-TKIs) targeting sensitizing mutations, such as L858R, has revolutionized the treatment paradigm for this subset of patients with advanced NSCLC, establishing these agents as the standard first-line therapeutic regimen (5). Both first- and second-generation agents (e.g., gefitinib, afatinib) and third-generation EGFR-TKIs (e.g., osimertinib) have consistently demonstrated superior progression-free survival (PFS) and objective response rates (ORR) compared with conventional chemotherapy (6, 7). However, despite marked initial efficacy, disease progression driven by acquired resistance remains virtually inevitable for all patients (8). Although third-generation EGFR-TKIs effectively overcome resistance mediated by the T790M mutation, the eventual emergence of more complex, non-T790M-dependent resistance mechanisms—typified by the C797S mutation, *Mesenchymal-Epithelial Transition factor* amplification, and *Human Epidermal Growth Factor Receptor 2* amplification—severely limits subsequent therapeutic options, posing a significant clinical challenge (9).

In recent years, immune checkpoint inhibitors (ICIs)—represented by inhibitors of programmed cell death protein 1 (PD-1) and programmed death-ligand 1 (PD-L1)—have achieved substantial success in the therapeutic landscape of NSCLC, significantly improving long-term survival in patient subsets lacking driver gene mutations (10). However, within the context of EGFR-mutated NSCLC, subgroup analyses from multiple phase III clinical trials indicate that patients do not derive benefit from ICI monotherapy in second-line or subsequent settings (11–13). Two meta-analyses have demonstrated that, compared with docetaxel, ICI monotherapy confers no advantage in overall survival (OS) for patients with EGFR-mutated NSCLC (14, 15). This lack of efficacy is generally attributed to the characteristic features of EGFR-mutated tumors, which include a lower tumor mutational burden (TMB), a paucity of tumor-infiltrating lymphocytes, and an immunosuppressive tumor microenvironment (TME) (16). Furthermore, emerging research suggests that significant heterogeneity exists within the immune microenvironment across distinct EGFR mutation subtypes. Compared with the 19-del subtype, the TME of L858R-mutated tumors appears phenotypically “colder, “ exhibiting lower PD-L1 expression levels, reduced CD8+ T cell infiltration, and a higher frequency of co-mutations that modulate immune responses, such as *TP53 (*17, 18*).* These biological disparities may render patients with the L858R mutation intrinsically more resistant to immunotherapy. Nevertheless, as findings remain inconsistent across studies, the critical importance of analyzing the L858R subtype independently is further underscored.

To surmount this intrinsic resistance to ICIs, researchers have investigated diverse combinatorial therapeutic strategies. Among these, the approach combining immunotherapy with anti-angiogenesis has attracted considerable interest. The mechanistic rationale underpinning this strategy is that anti-angiogenic agents (e.g., bevacizumab) not only inhibit tumor angiogenesis but also induce vascular normalization. This process facilitates the infiltration of effector T cells into the tumor parenchyma and downregulates vascular endothelial growth factor (VEGF)-mediated immunosuppression, thereby potentially converting immunologically “cold” tumors into “hot” ones and establishing a permissive microenvironment for ICI efficacy (19). Notably, the landmark IMpower150 trial demonstrated that among patients who experienced disease progression following EGFR-TKI therapy, the regimen of atezolizumab combined with bevacizumab and chemotherapy conferred a survival benefit compared with bevacizumab plus chemotherapy. Although these findings offer renewed promise for deploying immunotherapy in the EGFR-mutated, TKI-resistant population, they also raise concerns regarding additive toxicity and the precise identification of beneficiary subgroups (20).

Consequently, determining whether—and how—to implement immunotherapy in patients with EGFR-TKI-resistant, L858R-mutated NSCLC remains a contentious issue and a primary focus of clinical investigation. To address this knowledge gap, we conducted a systematic review and meta-analysis—the first to focus exclusively on the EGFR L858R mutation subtype—to comprehensively and quantitatively evaluate the ORR, PFS, and OS associated with distinct immunotherapeutic modalities (including ICI monotherapy, ICI plus chemotherapy, and ICI plus anti-angiogenic agents) following the failure of EGFR-TKI therapy. Our objective is to provide robust evidence to guide clinical decision-making for this specific patient population and to inform the design of future prospective clinical trials. It is important to note that this meta-analysis includes both randomized controlled trials (RCTs) and single-arm studies, which differ fundamentally in design and the type of evidence they provide. RCTs allow for direct comparison against a control group, yielding estimates of causal effect, whereas single-arm studies provide descriptive information on outcomes without a comparator. Therefore, pooled results from single-arm studies are presented primarily for exploratory purposes and should not be interpreted as definitive evidence of efficacy. Throughout this study, analyses were stratified by study design to account for these differences, and the implications of combining data from these heterogeneous sources are explicitly acknowledged.

## 2 Materials and methods

### 2.1 Search strategy

We conducted a systematic literature search in accordance with the Preferred Reporting Items for Systematic Reviews and Meta-Analyses (PRISMA) guidelines (21) and adhered to a pre-specified protocol. We systematically searched PubMed, EMBASE, the Cochrane Library, and Web of Science for prospective or retrospective clinical studies investigating immunotherapy in patients with EGFR-mutated NSCLC published up to January 1, 2026. The search was restricted to publications in the English language. Additionally, we performed manual searches of reference lists from relevant articles and reviewed abstracts or presentations from major conference proceedings, including the American Society of Clinical Oncology, the World Conference on Lung Cancer, the European Society for Medical Oncology, and the American Association for Cancer Research. Key search terms included: “non-small cell lung cancer, “ “immune checkpoint inhibitors, “ “tyrosine kinase inhibitors, “ and “epidermal growth factor receptor.” Following the removal of duplicate records, two investigators independently screened the remaining studies, initially at the title and abstract level, followed by a full-text review. Discrepancies were resolved through adjudication by a third investigator until consensus was achieved. This study protocol has been registered with the International Prospective Register of Systematic Reviews (PROSPERO) under registration number CRD420261278330.

### 2.2 Inclusion and exclusion criteria

Inclusion Criteria: Studies were included if they met the following criteria: (1) enrollment of patients with a confirmed diagnosis of EGFR-mutated NSCLC; (2) administration of an ICI-based treatment regimen in the experimental arm, including ICI monotherapy, ICI plus chemotherapy, or ICI combined with anti-angiogenic agents; (3) presence of a control arm receiving standard-of-care (e.g., chemotherapy, best supportive care), placebo, or an alternative ICI regimen; single-arm trials were also deemed eligible; (4) reporting of at least one of the following clinical outcomes: ORR, disease control rate (DCR), PFS, or OS (where PFS was defined as the time from randomization to the first documentation of disease progression or death, and OS was defined as the time from randomization to death from any cause); and (5) study design classified as a RCT or a non-randomized cohort study. Exclusion Criteria: Studies were excluded if they: (1) did not involve an immunotherapy intervention; (2) were reviews, case reports, letters, editorials, or preclinical animal studies; (3) contained incomplete data or indeterminate outcomes preventing effect size estimation; or (4) were published in a non-English language. In instances where multiple publications reported data from the same patient population, the most recent or comprehensive dataset was selected for analysis. Final study selection was performed by two authors, with any discrepancies resolved through discussion and consensus among the full investigative team.

### 2.3 Literature screening and data extraction

Initially, all retrieved citations were imported into EndNote reference management software for deduplication. Subsequently, two investigators independently performed a two-stage screening process. In the first phase (preliminary screening), titles and abstracts were reviewed to exclude clearly irrelevant records. In the second phase (full-text screening), the full texts of potentially eligible articles were assessed to determine final inclusion based on the pre-defined eligibility criteria. Throughout the screening process, any discrepancies between the two investigators were resolved through discussion; if consensus could not be reached, a third investigator adjudicated the final decision. Data extraction was similarly conducted independently by two investigators using a standardized, pre-designed Microsoft Excel spreadsheet. The following data were extracted: (1) baseline study characteristics (first author, year of publication, and study design); (2) baseline patient demographics; (3) specific treatment regimens for the intervention and control arms; and (4) outcome measures, including hazard ratios (HRs) with 95% confidence intervals (CIs), ORR, DCR, PFS, and OS.

### 2.4 Quality and bias assessment

The methodological quality of the included RCTs was evaluated using the Cochrane Risk of Bias Tool (22).This instrument assesses potential bias across seven distinct domains: (1) random sequence generation; (2) allocation concealment; (3) blinding of participants and personnel; (4) blinding of outcome assessment; (5) incomplete outcome data; (6) selective reporting; and (7) other potential sources of bias. For each domain, the risk was classified as “low, “ “high, “ or “unclear, “ and subsequently visualized in a risk of bias summary graph. The quality of non-randomized studies of interventions (NRSIs) was assessed using the MINORS (Methodological Index for Non-Randomized Studies) criteria (23).

### 2.5 Statistical analysis

Statistical analyses were conducted using Review Manager (RevMan) version 5.4 and R software version 4.5.1. For time-to-event outcomes (i.e., OS and PFS), HRs and their corresponding 95% CIs were utilized as the pooled effect measures. For dichotomous variables in single-arm studies, the DCR and its standard error were calculated for each study, and the pooled effect size was estimated using the pooled proportion method. Given the inclusion of both RCTs and non-randomized single-arm studies, pooled estimates were calculated separately by study design. HRs for OS and PFS were primarily derived from RCTs, while descriptive analyses of ORR and median survival times in single-arm studies are presented for exploratory purposes only, acknowledging the inherent limitations of non-comparative studies. Heterogeneity was assessed using the I² statistic and the Chi-square test, with a P-value < 0.05 indicating statistical significance. A random-effects model was employed for all analyses to account for between-study variance, given the substantial proportion of single-arm studies and observed heterogeneity. Publication bias was evaluated using funnel plots, complemented by Begg’s and Egger’s tests.Subgroup analyses were performed based on pre-specified factors, including intervention type and study design, as registered in the PROSPERO protocol. Due to the exploratory nature of some subgroup analyses, particularly those including a small number of studies or exhibiting high heterogeneity, findings should be interpreted with caution. Sensitivity analyses were subsequently performed to evaluate the robustness of pooled results. All statistical tests were two-sided, and a P-value < 0.05 was considered statistically significant.

## 3 Results

### 3.1 Literature search

**Figure 1:**
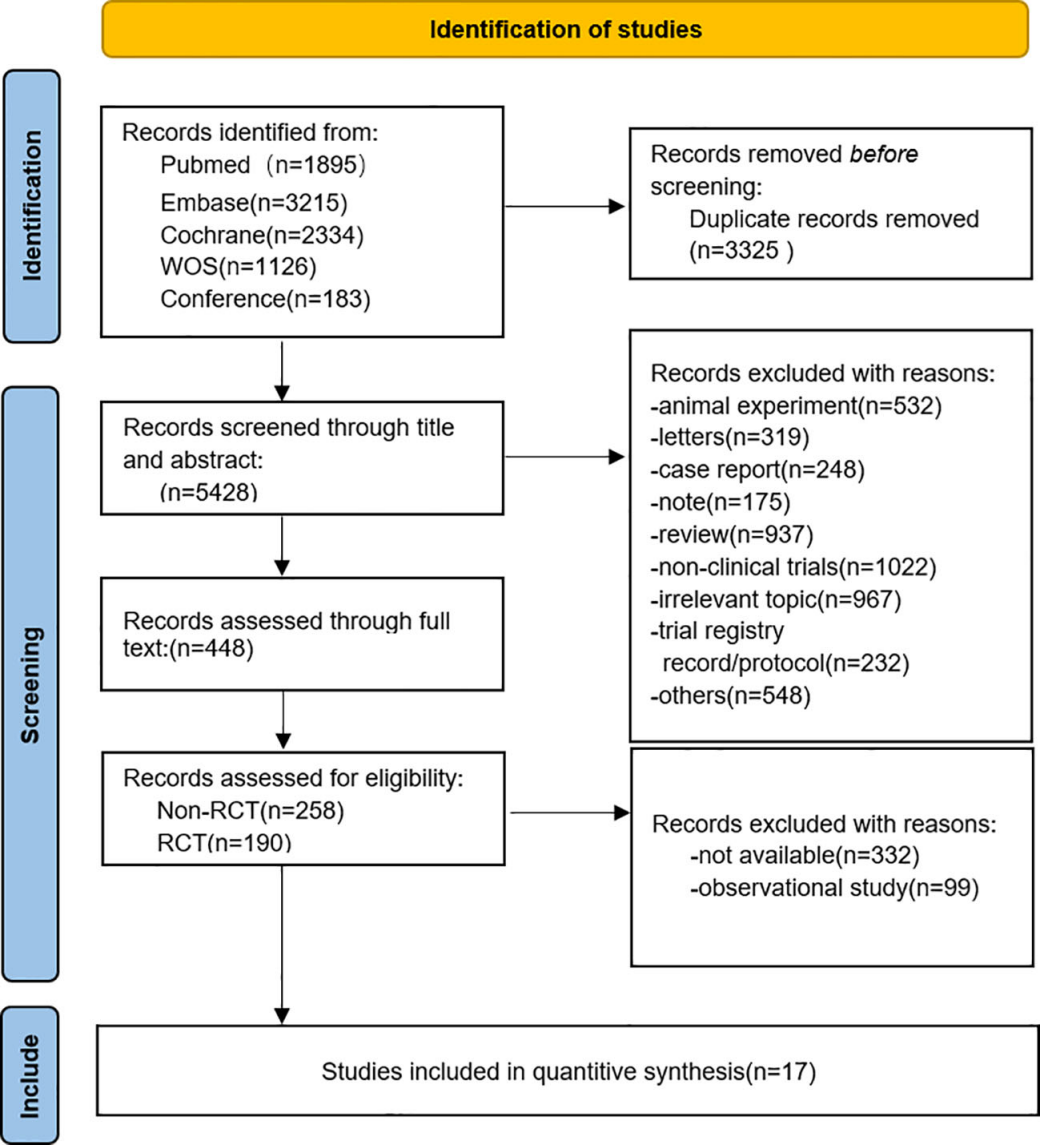
Flow diagram of the meta-analysis for inclusion and exclusion of studies.

A total of 8, 753 records were identified through the initial database search. Following the removal of 3, 325 duplicates, 1, 554 non-clinical or animal studies were excluded during the preliminary screening. An additional 967 studies were excluded after title and abstract review due to a lack of relevance to the study topic. Consequently, the full texts of the remaining 448 potentially eligible articles were comprehensively assessed. Ultimately, adhering to the specified inclusion and exclusion criteria, 17 studies were selected for the final analysis, comprising 6 RCTs (24–29)and 11 NRSIs (30–40).The detailed literature selection process is depicted in **Figure 1**.

### 3.2 Study characteristics

A total of 17 studies—encompassing 9 prospective (24–32)and 8 retrospective (33–40)analyses—were included in this meta-analysis. These cohorts, which comprised 5 phase II and 4 phase III clinical trials, involved a total of 1, 154 patients with EGFR L858R-mutated NSCLC receiving immunotherapy. The included studies were published between 2019 and 2025. Comprehensive baseline characteristics for each study are summarized in **Tables 1** and **2**.

**Table 1:** Baseline characteristics of the included randomized controlled trial studies.

Study	Year	Auther	Phase	Histology	Intervention	Control treatment	OS HR (95%CI)	PFS HR (95%CI)
IMpower151	2025	Zhou, C.	III	nsqNSCLC	Atezolizumab+Bev+CT (26)	Bev +CT(31)	1.01 (0.46, 2.23)	0.74(0.42-1.33)
ATTLAS	2024	Park, S.	III	nsqNSCLC	Atezolizumab+ Bev +CT(72)	CT(25)	1.12(0.60-2.10)	0.52(0.31-0.88)
HARMONi-A	2024	Fang, W.	III	nsqNSCLC	Ivonescimab+CT (60)	CT(78)	–	0.43(0.27-0.67)
KEYNOTE-789	2024	Yang, J. C. H.	III	nsqNSCLC	pembrolizumab+CT (103)	CT(102)	0.94(0.70-1.26)	0.86(0.64-1.17)
ORIENT-31	2023	Lu, S.	II	nsqNSCLC	Sintilimab+CT (62)	CT(61)	–	0·47 (0·31–0·72)
				nsqNSCLC	sintilimab+IBI305+CT (70)	CT(61)		0.37(0.24-0.57)
WJOG8515L	2021	Hayashi, H.	II	nsqNSCLC	Nivolumab (28)	CT(24)	0.97(0.48-1.93)	1.68(0.94-2.98)

CT, chemotherapy; nsq, non-squamous; PFS, progression-free survival; OS, overall survival; HR, hazard ratio; CI, confidence interval; Bev, Bevacizumab.

**Table 2:** Baseline characteristics of the included non-randomized controlled trial studies.

Auther	Year	Type of study	Histology	Intervention	ORR^a^, % (95%CI)	DCR^c^, % (95%CI)	mOS (95%CI), mo	mPFS (95%CI), mo
Lee, C. K (30).	2025	Prospective	nsqNSCLC	IO combination(27)	41.0(25.0-59.0)	88.9(72.6-96.9)	–	6.6(5.0–13.1)
Watanabe, S (31).	2024	Prospective	nsqNSCLC	IO combination(17)	–	–	–	8.0(5.7-11.0)
ATTLAS	2024	Prospective	nsqNSCLC	IO combination(72)	–	–	–	8.71(6.93-11.01)
Si, J (33).	2023	Retrospective	nsqNSCLC	IO combination(33)	24.2(11.1-42.3)	–	–	10.2(5.2-15.2)
Zhou, C (34).	2023	Retrospective	nsqNSCLC	IO/IO combination(51)	–	84.3	11.5(5.69–14.71)	6.4(5.64–7.17)
Zhong, H (32).	2023	Prospective	nsqNSCLC	IO combination(26)	57.7(38.4-75.1)	88.5(71.9-96.8)	–	10.1(5.7-14.9)
Hu, J (35).	2022	Retrospective	NSCLC	IO/IO combination(42)	16.7	52.4(36.9-67.5)	9.8(6.4-17.3)	2.5(1.9-5.0)
Morimoto, K (36).	2022	Retrospective	NSCLC	IO combination (60)	42.4(29.8-55.8)	81.8(70.1-90.2)	21.2(15.0-34.8)	7.0(5.6-8.5)
Guo, X (37).	2022	Retrospective	nsqNSCLC	IO/IO combination(29)		–	–	5.5(3.8-7.2)
Long, Y (38).	2021	Retrospective	NSCLC	IO combination (10)	40.0(12.2-73.8)	80.0(44.4-97.5)	11.6(9.6–13.6)	6.4(4.5–8.3)
Ito, T (39).	2021	Retrospective	NSCLC	IO(10)	30.0	50.0	14.8(0.00–34.61)	3.3(0.98-5.62)
Hastings, K (40).	2019	Retrospective	NSCLC	IO(46)	15.2(6.7-27.4)	36.9(23.2-52.2)	12.1(0.3–63)	–

nsq, non-squamous; PFS, progression-free survival; OS, overall survival; CI, confidence interval; ORR, overall response rate; DCR, disease control rate; IO, Immunotherapy.

### 3.3 Quality assessment of included studies

**Figure 2:**
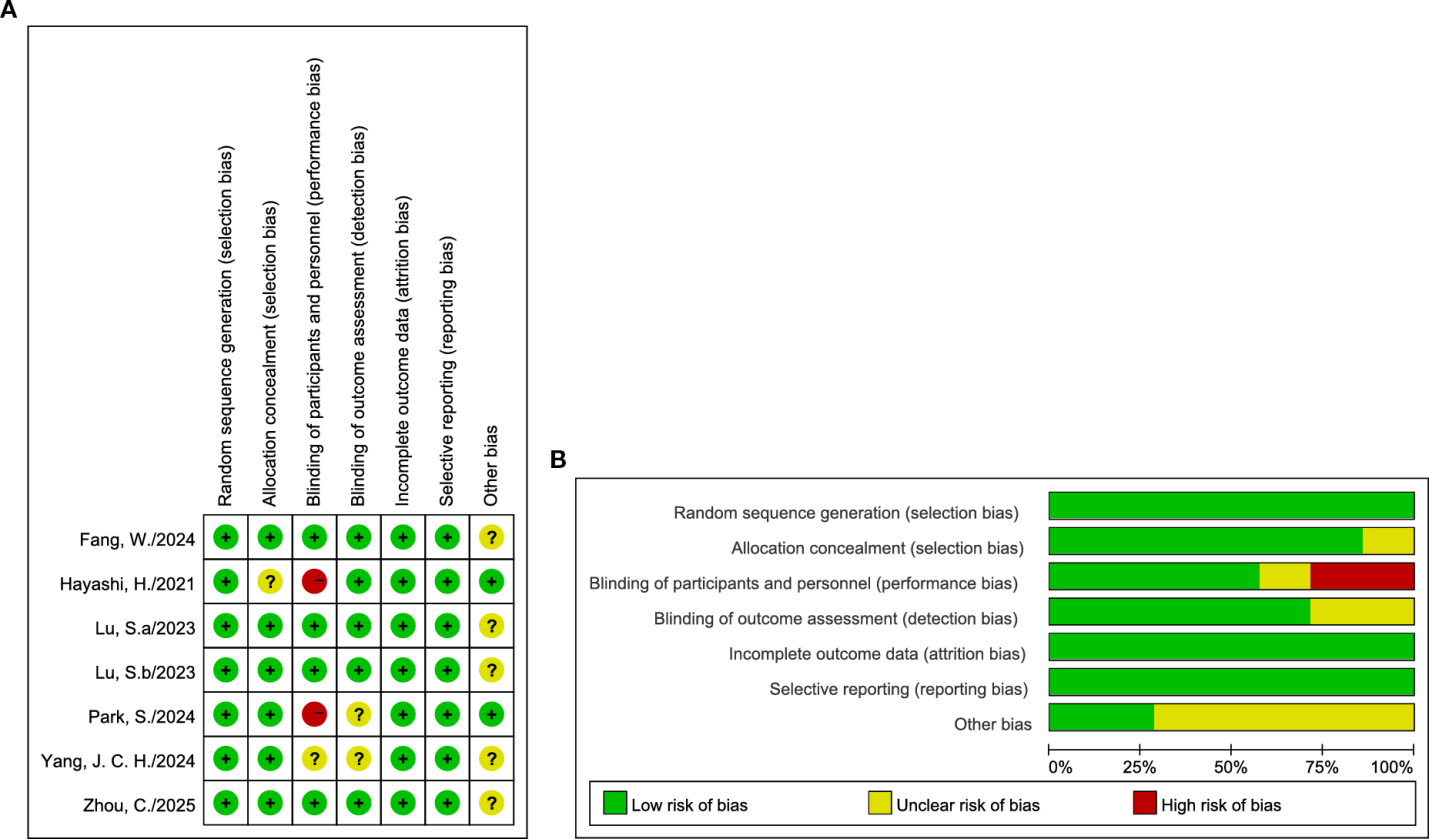
Quality assessment of the included studies. **(A)** The details of bias assessment. **(B)** The summary of bias assessment.

The methodological quality of the six included RCTs was evaluated using the Cochrane Risk of Bias Tool. Due to the open-label design inherent in several trials, blinding was not universally implemented. Additionally, specific studies were classified as having an “unclear” risk of bias attributed to limited data regarding sponsor involvement. Nevertheless, the overall risk of bias across the included studies was determined to be predominantly low (**Figure 2**). Given the heterogeneity of the single-arm cohorts, the MINORS tool was utilized to assess the quality of the 11 NRSIs, evaluating domains including study design, comparability, and outcome assessment, with detailed scores presented in **Supplementary Table 1**.

## 4 Meta-analysis results

### 4.1 Pairwise meta-analysis

**Figure 3:**
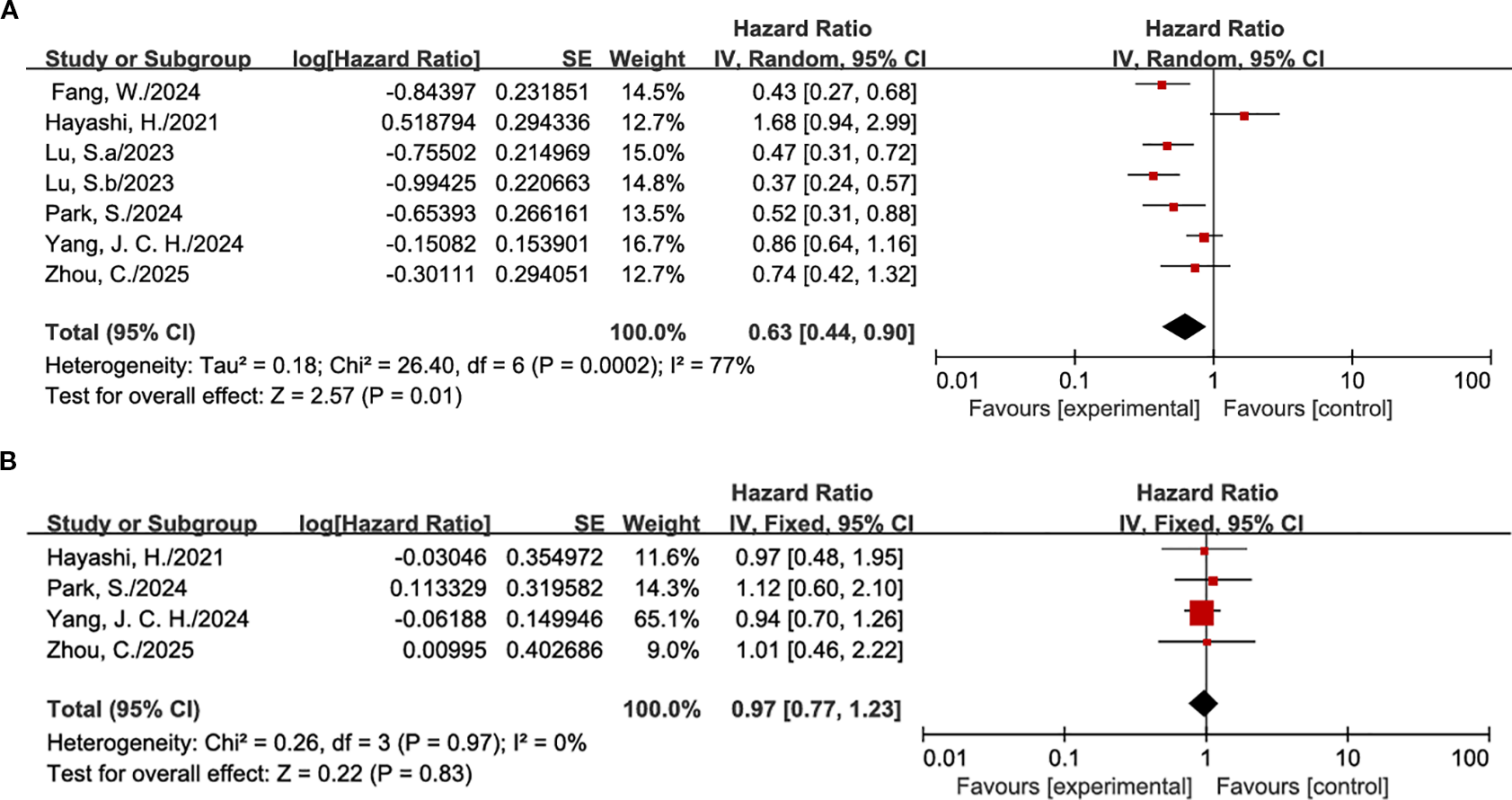
Forest plot comparing OS and PFS. **(A)** PFS; **(B)** OS.

Regarding PFS (**Figure 3A**), seven cohorts were included in the pairwise comparison. Pooled analysis using a random-effects model demonstrated that, compared with control regimens (predominantly chemotherapy), ICI-based therapies significantly improved PFS in patients with EGFR L858R mutations following TKI resistance (pooled HR = 0.63, 95% CI: 0.44–0.90, *P* = 0.01). However, substantial heterogeneity was observed across studies (*I²* = 77%). To elucidate the sources of this heterogeneity, subgroup analyses were conducted (**Supplementary Figure 1A**). The results indicated that the PFS benefit was driven primarily by combinatorial therapeutic strategies: specifically, both ICI plus chemotherapy (HR = 0.57) and ICI plus anti-angiogenic agents (HR = 0.50) demonstrated significant efficacy. In contrast, ICI monotherapy failed to confer a PFS benefit (HR = 1.68). Tests for subgroup differences revealed statistically significant disparities among the distinct treatment modalities (*P* = 0.002). Leave-one-out sensitivity analyses were performed to assess the robustness of pooled PFS estimates (**Supplementary Figure 2A**). Overall, sequential exclusion of individual studies did not materially alter the pooled hazard ratio; however, removal of KEYNOTE−789 or WJOG8515L produced notable shifts in the pooled estimate. Specifically, exclusion of KEYNOTE−789 slightly decreased heterogeneity but attenuated the pooled PFS benefit, which likely reflects its relatively large sample size and that it enrolled patients with EGFR−mutated NSCLC who had progressed on prior EGFR−TKI therapy, receiving pembrolizumab plus chemotherapy as a post-TKI treatment regimen. In contrast, exclusion of WJOG8515L increased the pooled PFS estimate and reduced statistical significance, attributable to its small sample size and single-agent nivolumab intervention, representing a less intensive immunotherapy approach compared with other combination regimens included in the meta-analysis. These observations indicate that both studies exert disproportionate influence on the overall meta-analytic outcome, contributing to the high heterogeneity observed, and highlight the importance of considering differences in sample size, treatment intensity, and prior EGFR-TKI exposure when interpreting the robustness of pooled PFS results. Assessment of publication bias via funnel plot inspection (**Supplementary Figure 3A**) revealed a generally symmetrical distribution. Begg’s and Egger’s tests were subsequently employed to quantitatively evaluate publication bias; both tests indicated no evidence of significant bias (*P* > 0.05), with detailed results presented in **Supplementary Table 2**.

For the OS endpoint (**Figure 3B**), data suitable for pairwise comparison were available from four studies. Given the absence of heterogeneity across these studies, a fixed-effects model was employed. The pooled analysis indicated that, compared with control arms, current ICI-based regimens did not confer a statistically significant OS benefit in patients harboring the EGFR L858R mutation (pooled HR = 0.97, 95% CI: 0.77–1.23, *P* = 0.83). In subgroup analyses (**Supplementary Figure 1B**), none of the specific immunotherapeutic strategies—including monotherapy, combination with chemotherapy, or combination with anti-angiogenic agents plus chemotherapy—yielded an OS significantly superior to that of the control arm, nor were statistically significant differences observed between subgroups. Sensitivity analysis (**Supplementary Figure 2B**) confirmed the robustness of these findings, demonstrating that the pooled estimates remained stable following the sequential exclusion of individual studies. Furthermore, visual inspection of the funnel plot (**Supplementary Figure 3B**) revealed substantial symmetry, suggesting an absence of overt publication bias. This was corroborated by both Begg’s and Egger’s tests, which confirmed the lack of significant bias; detailed results are presented in **Supplementary Table 2**.

### 4.2 Single-arm meta-analysis

**Figure 4:**
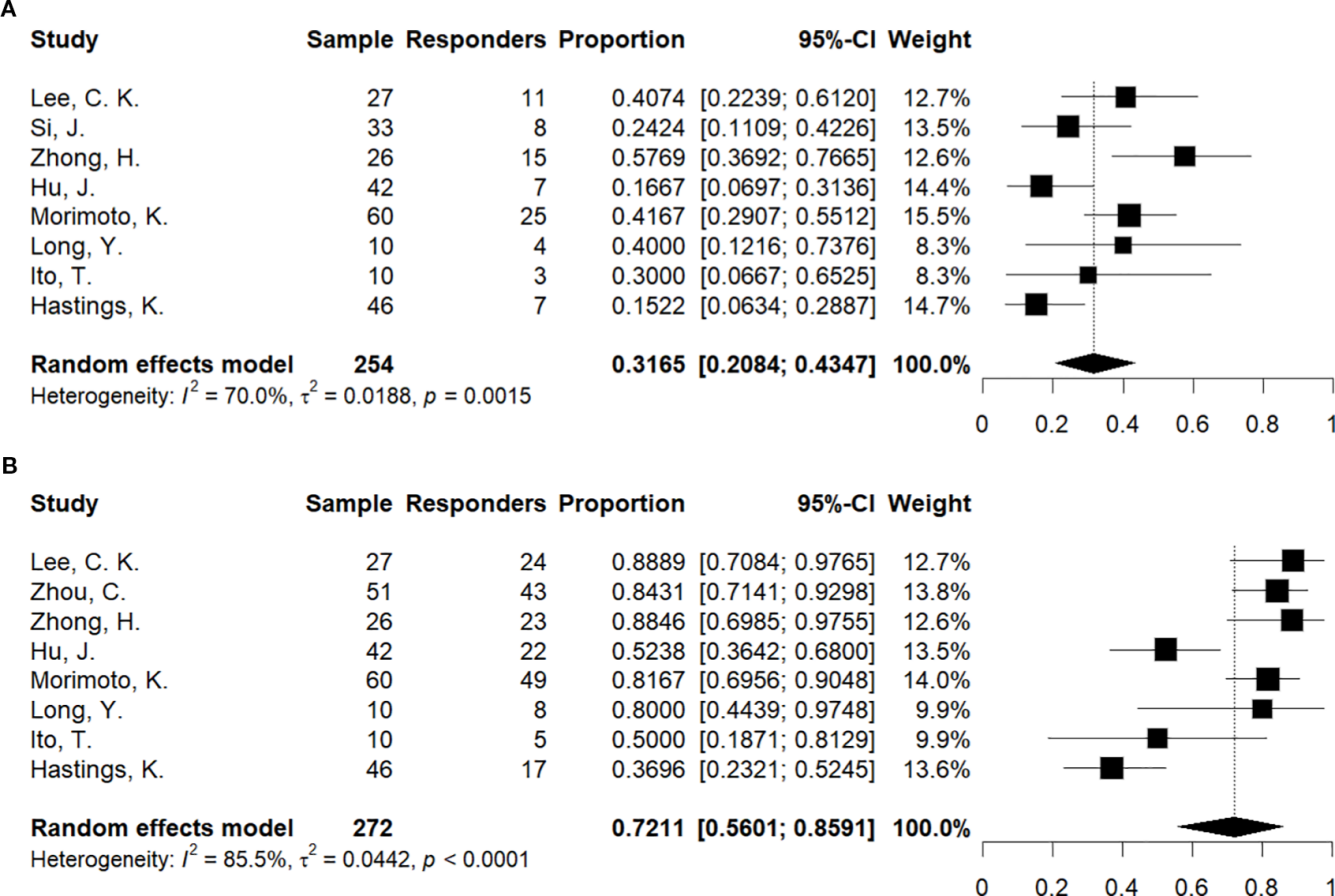
Forest plot summarizing the single-arm ORR and DCR. **(A)** ORR; **(B)** DCR.

In the single-arm meta-analysis evaluating ORR in the target population (**Figure 4A**), eight studies comprising 254 patients were included. Due to substantial heterogeneity among studies, a random-effects model was utilized, yielding a pooled ORR of 31.7% (95% CI: 20.8%–43.5%, *P* = 0.0015). Subgroup analyses revealed that both intervention modality and study design significantly influenced outcomes (**Supplementary Figures 4A**, **5A**). The pooled ORR for immunotherapy-based combination regimens (40.2%) was significantly higher than that for monotherapy (17.6%). Furthermore, the pooled ORR reported in prospective studies (49.1%) was significantly superior to that observed in retrospective cohorts (25.9%). Differences between these subgroups were statistically significant (*P* = 0.0057 and *P* = 0.0229, respectively). Regarding publication bias, the funnel plot (**Supplementary Figure 6A**) exhibited no obvious asymmetry. Consistent with this observation, neither Begg’s nor Egger’s tests indicated significant publication bias, confirming the reliability of these findings. Detailed results of the bias assessments are provided in **Supplementary Table 2**.

For the DCR analysis (**Figure 4B**), eight studies involving 272 patients were included. Individual study DCR estimates ranged from 36.96% to 88.89%. Due to pronounced heterogeneity across studies, a random-effects model was employed, yielding a pooled overall DCR of 72.1% (95% CI: 56.0%–85.9%, *P* < 0.0001). Subgroup analyses (**Supplementary Figures 4B**, **5B**) identified significant disparities based on treatment modality and study design: the pooled DCR for immunotherapy-based combination regimens (85.2%) was significantly higher than for monotherapy (38.9%), and prospective studies reported a significantly superior DCR (88.7%) compared with retrospective cohorts (65.5%). These differences were statistically significant in both subgroup comparisons. Collectively, these data indicate an overall DCR of 72.1% in the target population, with intervention modality and study design serving as key determinants of efficacy. Regarding publication bias, the funnel plot (**Supplementary Figure 6B**) displayed no marked asymmetry. This was corroborated by both Begg’s and Egger’s tests, which indicated no evidence of significant bias; detailed results are provided in **Supplementary Table 2**.

**Figure 5:**
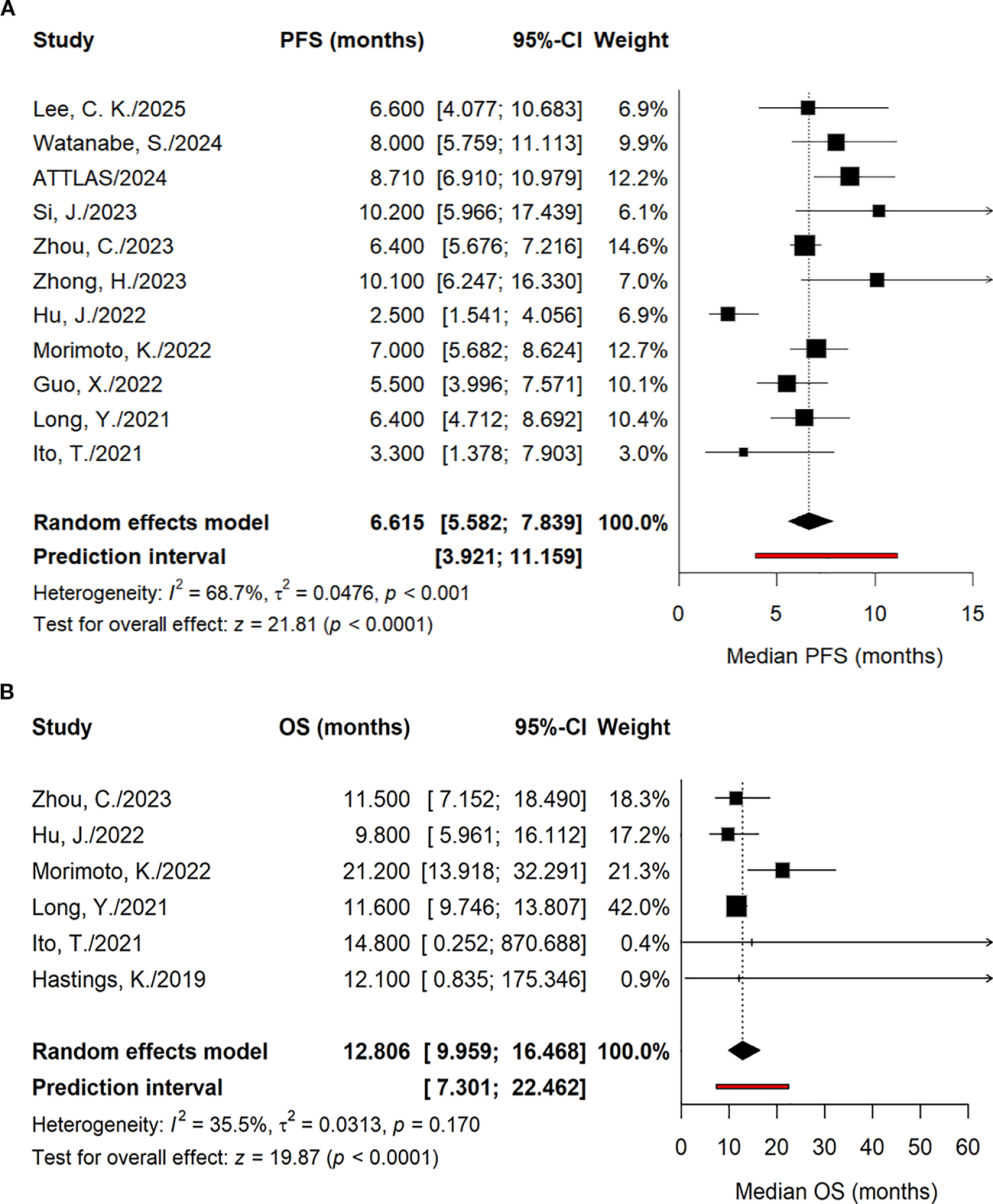
Forest plot summarizing the single-arm PFS and OS. **(A)** PFS; **(B)** OS.

A single-arm meta-analysis was performed for PFS (**Figure 5A**), incorporating data from 11 studies. The median PFS reported in individual cohorts ranged from 2.50 to 10.20 months. Given the moderate heterogeneity observed across studies, a random-effects model was employed, yielding a pooled median PFS of 6.615 months (95% CI: 5.582–7.839, *P* < 0.0001). Subgroup analyses (**Supplementary Figures 7A**, **8**) revealed that the ICI combination regimen subgroup demonstrated a significantly superior median PFS compared with the ICI monotherapy subgroup. Furthermore, the pooled median PFS observed in prospective studies was significantly longer than that in retrospective cohorts. These subgroup differences were statistically significant (*P* = 0.0214 and *P* = 0.0083, respectively). Collectively, these results indicate a pooled median PFS of 6.615 months for the target population, with treatment modality and study design identified as key determinants of outcomes. The funnel plot (**Supplementary Figure 9A**) displayed no marked asymmetry, and neither Begg’s nor Egger’s tests indicated significant publication bias, confirming the robustness of these findings. Detailed results of the bias assessments are presented in **Supplementary Table 2**.

For the OS analysis (**Figure 5B**), six studies were included. Median OS estimates reported in individual cohorts ranged from 9.80 to 21.20 months. Given the mild heterogeneity observed across studies, the pooled median OS was calculated as 12.806 months (95% CI: 9.959–16.468), with a prediction interval of 7.301–22.462 months. Subgroup analyses (**Supplementary Figure 7B**) revealed that, although the median OS for the ICI combination regimen subgroup was numerically superior to that of the dual ICI and ICI monotherapy subgroups, these differences did not reach statistical significance. Collectively, these data indicate a pooled median OS of 12.806 months for the target population, with no statistically significant variance observed across distinct intervention modalities. Regarding publication bias, the funnel plot (**Supplementary Figure 9B**) exhibited no marked asymmetry. This was corroborated by both Begg’s and Egger’s tests, neither of which indicated significant bias; detailed results are provided in **Supplementary Table 2**.

## 5 Discussion

Through a systematic meta-analysis of 17 clinical cohorts comprising 1, 154 patients, we quantitatively evaluated the therapeutic utility of ICIs in patients with EGFR L858R-mutated NSCLC following TKI resistance. Our principal findings indicate that, while ICI monotherapy exhibits suboptimal efficacy in this subtype, combinatorial strategies incorporating chemotherapy or anti-angiogenic agents significantly improve PFS and ORR. Several key analyses demonstrated high heterogeneity, and multiple subgroup analyses were conducted. We acknowledge that multiple comparisons may increase the risk of false-positive findings, and some subgroup comparisons involved a small number of studies, reducing statistical reliability. The selection of certain subgroup stratifications (e.g., intervention type versus study design) was based on clinical rationale; however, interpretation of these findings should remain cautious. Future studies with larger sample sizes and pre-specified subgroup analyses are warranted to confirm these exploratory observations. These results not only underscore the critical importance of modulating the TME to overcome immune resistance associated with EGFR mutations but also provide a robust evidence-based framework for precision medicine targeting the specific L858R subtype. Notably, although PFS benefits are evident, our analysis confirms that OS improvements were not statistically significant either overall or within subgroups. This dissociation may be influenced by crossover effects, subsequent lines of therapy, and treatment-related toxicities, all of which can dilute OS signals. Consequently, while combination therapy demonstrates clear advantages in delaying disease progression, it does not yet provide definitive evidence of survival extension. Therefore, the clinical interpretation should emphasize improved disease control rather than conclusive survival benefit.

Initially, our analysis reaffirms that ICI monotherapy lacks clinical utility for patients harboring the EGFR L858R mutation. This conclusion aligns with earlier observations by Gainor et al. (41, 42). As early as 2017, the seminal meta-analysis by Lee et al. (14) established the paradigm that patients with EGFR mutations generally fail to derive survival benefit from ICI monotherapy, a finding that provided critical guidance for clinical practice. Our study specifically corroborates this conclusion within the L858R subtype, confirming that exclusive blockade of the PD-1/PD-L1 axis is insufficient to overcome the immunosuppressive microenvironment driven by the L858R mutation. However, with the evolution of combinatorial strategies, a recent systematic review by Qian et al. (43) indicated that ICI-based combination therapies confer significant PFS and ORR benefits in the EGFR-TKI-resistant population. Unlike Qian et al., who analyzed 19-del and L858R mutations in aggregate, our study focused exclusively on the L858R subtype. We observed that the response of this subtype to combination therapy is comparable to—and for certain regimens, potentially more pronounced than—that of the overall EGFR-mutant population. This suggests that patients with L858R mutations are not universally refractory to immunotherapy; rather, efficacy depends on identifying the optimal combinatorial partners. It should be noted that, although this study specifically focused on the L858R mutation, several included trials were not originally powered or designed to detect L858R-specific effects. Many subgroup analyses were *post hoc* and may be incomplete or subject to reporting bias. Furthermore, no direct statistical interaction testing was conducted between L858R and exon 19 deletion subgroups. As a result, it remains uncertain whether the observed efficacy of combination therapy truly reflects biological differences specific to L858R tumors or is influenced by treatment intensity effects observed across the broader EGFR-mutant population. Therefore, conclusions regarding L858R-specific sensitivity should be interpreted with caution and warrant validation in prospective, subtype-targeted trials. Notably, our findings parallel a recent landmark meta-analysis by Zhao et al. (44), which identified that ICI combined with anti-angiogenic agents and chemotherapy (the “four-drug regimen”) represents the optimal therapeutic strategy following TKI resistance. Furthermore, their subgroup analysis revealed a notable trend wherein patients with L858R mutations appeared to derive greater PFS benefit from the four-drug regimen compared with those harboring 19-del mutations. By pooling data from both single-arm and controlled studies, our analysis specifically validates this observation within the L858R population, demonstrating superior outcomes in patients treated with ICIs combined with anti-angiogenic agents. This finding provides statistical substantiation for the hypothesis proposed by Zhao et al., suggesting that L858R-mutated tumors may possess distinct biological characteristics that render them particularly sensitive to the synergistic effects of immunotherapy and anti-angiogenic blockade.

Mechanistically, EGFR mutation-driven tumors typically exhibit an “immune desert” or “immune-excluded” phenotype. The seminal work by Akbay et al. demonstrated that constitutive activation of the EGFR signaling pathway can upregulate PD-L1 expression; however, this upregulation is driven not by T-cell-mediated inflammation but by oncogenic signaling that paradoxically induces T-cell apoptosis (45). Furthermore, compared with wild-type tumors or those harboring alternative driver alterations, EGFR-mutated malignancies generally possess a lower TMB. This deficiency compromises the effective presentation of neoantigens, thereby impeding the recruitment and infiltration of CD8+ cytotoxic T lymphocytes into the tumor parenchyma (46, 47). Consequently, exclusive blockade of the PD-1/PD-L1 axis proves insufficient to reverse this profound immunosuppressive state. Furthermore, the disparity in immunogenicity between the two canonical mutations, L858R and 19-del, remains a subject of academic debate. A study by Hastings et al. previously suggested that L858R-mutated tumors exhibit a marginally higher TMB compared with 19-del tumors and numerically appear to demonstrate improved response rates to PD-L1 inhibitors (40). However, our meta-analysis failed to identify a significant clinical benefit for the L858R subtype treated with immunotherapy alone. This discrepancy may be attributable to distinct co-mutational landscapes frequently associated with the L858R allele. For instance, genomic profiling by Skoulidis et al. revealed that L858R mutations frequently co-occur with alterations in genes governing epigenetic regulation (e.g., *DNMT3A*) or cell cycle control; such co-mutations may further dampen anti-tumor immunity via non-TMB-dependent mechanisms (16, 18, 48). This implies that future research must move beyond stratifying patients solely by EGFR mutation subtype, integrating multi-omics sequencing to identify specific L858R subpopulations that are genuinely poised to benefit from immunotherapy.

Given the inherent limitations of monotherapy, combinatorial therapeutic strategies have emerged as a pivotal breakthrough. Our analysis demonstrates that “four-drug” or “three-drug” regimens incorporating anti-angiogenic agents (e.g., ICI + bevacizumab + chemotherapy) yield the most significant PFS benefit. This finding is highly consistent with results from the landmark IMpower150 trial and its subsequent counterpart in the Chinese population, ORIENT-31 (20, 28). In these combinatorial regimens, anti-angiogenic agents such as bevacizumab are hypothesized to function as immune sensitizers based on preclinical and clinical literature. Although the included studies did not directly evaluate changes in the tumor microenvironment or VEGF-mediated immunosuppression, prior reports suggest that anti-angiogenic therapy may normalize tumor vasculature and facilitate T cell infiltration (49). Therefore, the potential conversion of ‘cold’ tumors into ‘hot’ ones and downregulation of immunosuppressive populations such as myeloid-derived suppressor cells and regulatory T cells remains a theoretical mechanism rather than a finding directly supported by our meta-analytic data. For patients harboring the L858R mutation, this hypothesized remodeling of the tumor microenvironment may contribute to observed PFS benefits, but our meta-analysis cannot confirm this mechanistic effect. However, it must be explicitly acknowledged that despite the significant prolongation of PFS, the OS benefit observed in this study did not reach statistical significance. This outcome parallels the findings of two recently published landmark phase III clinical trials—KEYNOTE-789 (pembrolizumab plus chemotherapy) and CheckMate 722 (nivolumab plus chemotherapy)—neither of which demonstrated a significant improvement in OS within the EGFR-mutated population (27, 50). The dissociation between PFS and OS may be attributable to multidimensional factors: first, the crossover effect of subsequent lines of therapy may dilute the initial survival benefit; second, immune-related adverse events associated with combination immunotherapy may offset the survival advantages conferred by anti-tumor activity; and third, patients with EGFR L858R mutations often exhibit substantial tumor heterogeneity following TKI resistance. A subset of these patients may undergo rapid histological transformation to small cell lung cancer or develop bypass activation mechanisms, such as *Mesenchymal-Epithelial Transition factor* amplification, thereby hindering the maintenance of long-term disease control via immunotherapy and chemotherapy alone (51).

Although this study substantiates the potential of ICI-based combination therapy to improve PFS, the translation of this PFS benefit into a definitive prolongation of OS remains a primary challenge for future investigation. Given that conventional “immunotherapy plus chemotherapy” strategies may have reached a therapeutic plateau, future explorations should prioritize the following three directions: First, the incorporation of novel pharmacological classes. Antibody-drug conjugates (ADCs) are reshaping the therapeutic landscape of EGFR-mutated NSCLC. For instance, the HER3-targeted agent patritumab deruxtecan has demonstrated encouraging anti-tumor activity in patients with EGFR-TKI resistance, independent of specific EGFR resistance mechanisms (52). Future clinical trials should investigate whether combinatorial modalities such as “ICI plus ADC” or “TKI plus ADC” can yield efficacy superior to existing chemotherapy-based regimens specifically within the L858R subtype.Second, precise stratification based on co-mutation profiles. The L858R mutation is not a monolithic entity; it frequently co-occurs with mutations in genes such as *TP53*, *RB1*, or *STK11*, which profoundly influence the immune microenvironment and therapeutic sensitivity (53). Future clinical trials should not rely solely on EGFR subtype stratification but must integrate multi-gene next-generation sequencing to explore differential responses to composite biomarkers, thereby realizing the goal of truly precision immunotherapy.Third, the application of liquid biopsy for dynamic monitoring. Given the challenges associated with repeat tissue biopsies, the adoption of circulating tumor DNA (ctDNA)-based dynamic monitoring should be promoted. Monitoring ctDNA clearance (blood TMB) or molecular response may predict long-term immunotherapeutic benefits earlier than radiographic imaging, thereby guiding timely therapeutic adjustments or treatment de-escalation (54).

This study presents several limitations that merit consideration when interpreting the results. It should be noted that a substantial portion of included studies were non-randomized single-arm trials. While these studies contribute valuable descriptive information regarding response rates and median survival, they are subject to inherent selection and reporting biases. Therefore, pooled estimates from single-arm studies are presented for exploratory purposes only and should not be interpreted as definitive evidence of treatment efficacy. First, as a meta-analysis based on published literature, we utilized aggregate data rather than individual patient data. This restricted our ability to adjust for potential confounding variables—such as performance status, smoking history, and the number of prior TKI therapy lines—and precluded the precise reconstruction of survival curves for the L858R subgroup, thereby limiting the granularity of time-to-event analyses. Second, substantial heterogeneity exists among the included studies. Our analysis synthesized data from both RCTs and single-arm retrospective cohorts. Although a random-effects model was employed to address this heterogeneity and sensitivity analyses confirmed the robustness of our findings, the inclusion of retrospective data inevitably introduces inherent selection and information biases. Third, despite our specific focus on the L858R subtype, sample sizes for certain subgroup analyses—particularly those regarding OS—remained limited. Follow-up durations varied substantially across studies, and the high rate of crossover to immunotherapy in control arms upon disease progression may have confounded the OS analysis, potentially diluting the survival benefit associated with combination therapies. Furthermore, it is important to acknowledge that several included studies were not originally powered for L858R-specific analyses. Consequently, subgroup results may be influenced by *post hoc* analyses, incomplete reporting, or potential publication bias. Moreover, no formal statistical interaction testing between L858R and exon 19 deletion subgroups was conducted. Therefore, while combinatorial therapies appear effective in the L858R population, it remains uncertain whether these benefits are truly subtype-specific or reflect broader effects observed across EGFR-mutant NSCLC cohorts. Consequently, interpretation of the OS data warrants caution, underscoring the necessity for more mature, long-term follow-up data.

In summary, for patients with TKI-resistant, EGFR L858R-mutated NSCLC, ICI monotherapy lacks efficacy and should be avoided. Evidence from RCTs indicates that combinatorial strategies—particularly “immunotherapy plus anti-angiogenesis plus chemotherapy”—may improve PFS. Although single-arm studies provide supportive descriptive data, these findings should be interpreted cautiously due to inherent study limitations. However, translating the PFS advantage into a definitive prolongation of OS remains a significant challenge. Future research should focus on developing combinatorial therapies targeting novel immune checkpoints and exploring dynamic biomarkers via liquid biopsy, with the ultimate goal of precisely identifying L858R-mutated subpopulations capable of deriving long-term benefit from combination immunotherapy.

## 6 Conclusion

Through a systematic review and meta-analysis specifically targeting patients with EGFR L858R-mutated NSCLC, this study provides evidence-based guidance for therapeutic decision-making in this refractory subgroup following TKI resistance. Our analysis confirms that, due to the immunosuppressive tumor microenvironment, ICI monotherapy lacks clinical efficacy in patients harboring the L858R mutation and should not be routinely recommended. In contrast, ICI-based combination therapies—particularly the four-drug regimen comprising ICI plus anti-angiogenic agents plus chemotherapy—demonstrate significant PFS and ORR benefits. These findings support the hypothesis that the L858R subtype may exhibit heightened sensitivity to the anti-angiogenesis plus immunotherapy approach, which may be related to its putative biological characteristics suggested by prior studies. However, given the lack of statistically significant OS improvement, these results should be interpreted as evidence of enhanced disease control rather than definitive survival extension. Future research should prioritize novel therapeutic modalities, such as antibody-drug conjugates and bispecific antibodies, and incorporate co-mutation profiling and liquid biopsy technologies to enable precise stratification of beneficiary populations, ultimately aiming to achieve long-term survival benefits in patients with the L858R mutation.

## Data Availability

The original contributions presented in the study are included in the article/**Supplementary Material**. Further inquiries can be directed to the corresponding author.
