# Supplementary material for: Immunotherapy for TKI-resistant, EGFR L858R-mutated non-small cell lung cancer: a systematic review and meta-analysis of randomized and single-arm studies
Source: Front Immunol. 2026 Apr 10;17:1787310. doi: 10.3389/fimmu.2026.1787310 (PMC13106209; doi:10.3389/fimmu.2026.1787310)
Supplement: Supplementary file 1 [file DataSheet1.zip › Supplementary Material Presentation/Abbreviations.docx]

immune checkpoint inhibitors (ICIs); epidermal growth factor receptor (EGFR); non-small cell lung cancer (NSCLC); tyrosine kinase inhibitor (TKI); progression-free survival (PFS); objective response rate (ORR); overall survival (OS); hazard ratios (HRs); confidence intervals (CI); 19 deletion (19-del); EGFR tyrosine kinase inhibitors (EGFR-TKIs); programmed cell death protein 1 (PD-1); programmed death-ligand 1 (PD-L1); tumor mutational burden (TMB); tumor microenvironment (TME); vascular endothelial growth factor (VEGF); disease control rate (DCR); randomized controlled trial (RCT); Newcastle-Ottawa Scale (NOS); non-randomized studies of interventions (NRSIs); Antibody-drug conjugates (ADCs); circulating tumor DNA (ctDNA).
