# Supplementary material for: Immunotherapy for TKI-resistant, EGFR L858R-mutated non-small cell lung cancer: a systematic review and meta-analysis of randomized and single-arm studies
Source: Front Immunol. 2026 Apr 10;17:1787310. doi: 10.3389/fimmu.2026.1787310 (PMC13106209; doi:10.3389/fimmu.2026.1787310)
Supplement: Supplementary file 1 [file DataSheet1.zip › Supplementary Material Presentation/Supplementary Figure and Table.docx]

Table S1 Quality assessment of included studies by MINORS.

| Study | 1 | 2 | 3 | 4 | 5 | 6 | 7 | 8 | 9 | 10 | 11 | 12 | Total |
| --- | --- | --- | --- | --- | --- | --- | --- | --- | --- | --- | --- | --- | --- |
| Lee, C. K./2025 | 2 | 2 | 2 | 2 | 0 | 2 | 2 | 2 | 0 | 0 | 0 | 2 | 16 |
| Watanabe, S./2024 | 2 | 2 | 2 | 2 | 2 | 2 | 2 | 2 | 0 | 0 | 0 | 2 | 18 |
| Si, J./2023 | 2 | 0 | 2 | 2 | 1 | 2 | 1 | 0 | 0 | 0 | 0 | 2 | 12 |
| Zhou, C./2023 | 2 | 1 | 0 | 2 | 1 | 2 | 2 | 0 | 0 | 0 | 0 | 2 | 12 |
| Zhong, H./2023 | 2 | 1 | 2 | 2 | 1 | 2 | 2 | 2 | 0 | 0 | 0 | 2 | 16 |
| Hu, J./2022 | 2 | 1 | 0 | 2 | 2 | 2 | 2 | 0 | 0 | 0 | 0 | 2 | 13 |
| Morimoto, K./2022 | 2 | 1 | 0 | 2 | 1 | 2 | 2 | 0 | 2 | 2 | 1 | 2 | 17 |
| Guo, X./2022 | 2 | 1 | 0 | 2 | 1 | 2 | 2 | 0 | 0 | 0 | 0 | 2 | 12 |
| Long, Y./2021 | 2 | 1 | 0 | 2 | 1 | 2 | 2 | 0 | 2 | 2 | 1 | 2 | 17 |
| Ito, T./2021 | 2 | 1 | 0 | 2 | 1 | 2 | 2 | 0 | 0 | 0 | 0 | 2 | 12 |
| Hastings, K./2019 | 2 | 1 | 0 | 2 | 1 | 2 | 2 | 0 | 0 | 0 | 0 | 2 | 12 |

1.A stated aim of the study; 2.Inclusion of consecutive patients; 3.Prospective collection of data; 4.End point appropriate to the study aim; 5.Unbiased evaluation of endpoints; 6.Follow-up period appropriate to the major endpoint; 7.Loss to follow up not exceeding 5%; 8.Prospective calculation of the sample size; 9.A control group having the gold standard intervention; 10.Contemporary groups; 11.Baseline equivalence of groups; 12.Statistical analyses adapted to the study design.

Abbreviations: Methodological index for non-randomized studies (MINORS)

Table S2 P value of Begg’s test and Egger’s test.

|  | single-arm ORR | single-arm DCR | single-arm PFS | single-arm OS | PFS | OS |
| --- | --- | --- | --- | --- | --- | --- |
| Begg’s test | 0.383 | 0.708 | 0.586 | 0.348 | 0.368 | 0.734 |
| Eegg’s test | 0.589 | 0.991 | 0.759 | 0.726 | 0.958 | 0.313 |

Abbreviations: progression-free survival (PFS); overall survival (OS); overall response rate (ORR); disease control rate (DCR).

**A.
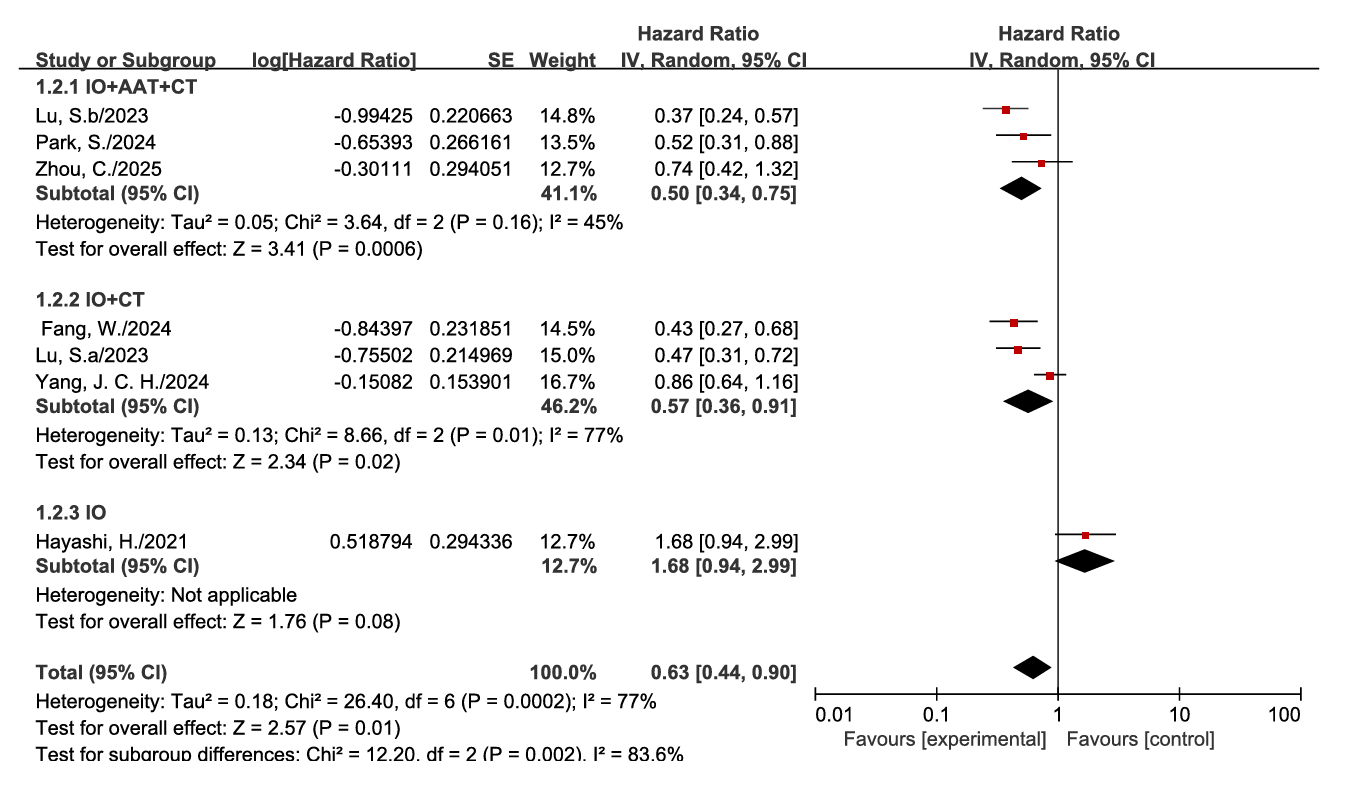
B.
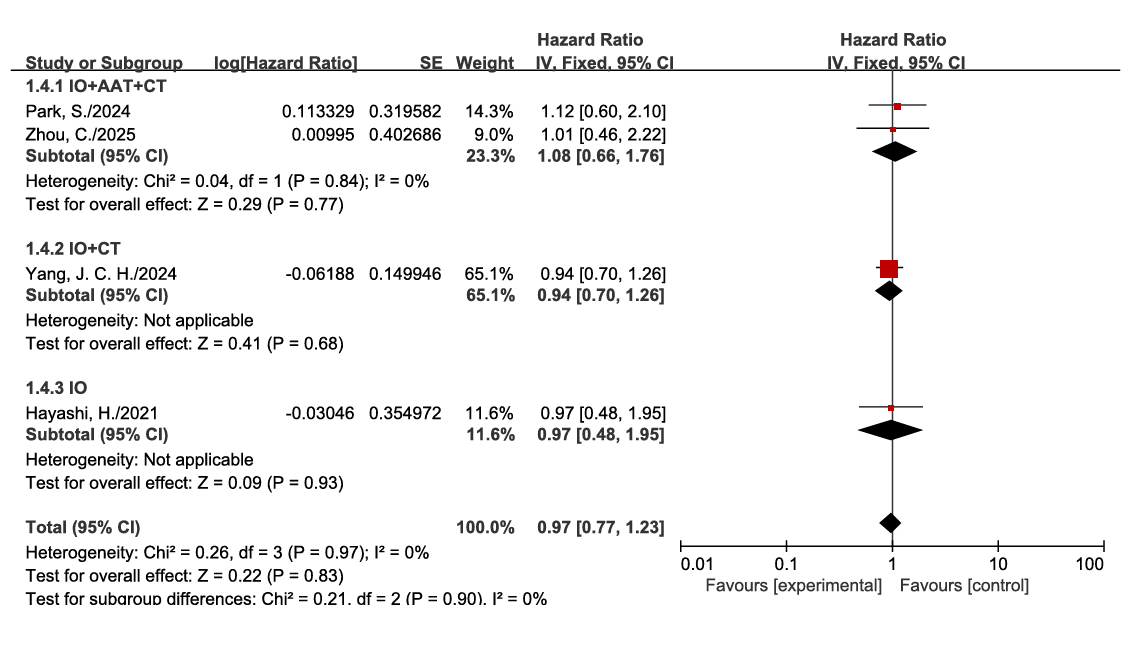
**

**Figure S1 Forest plot of subgroup analysis based on different treatment approaches. (A) PFS; (B) OS. progression-free survival (PFS); overall survival (OS).**

**A.
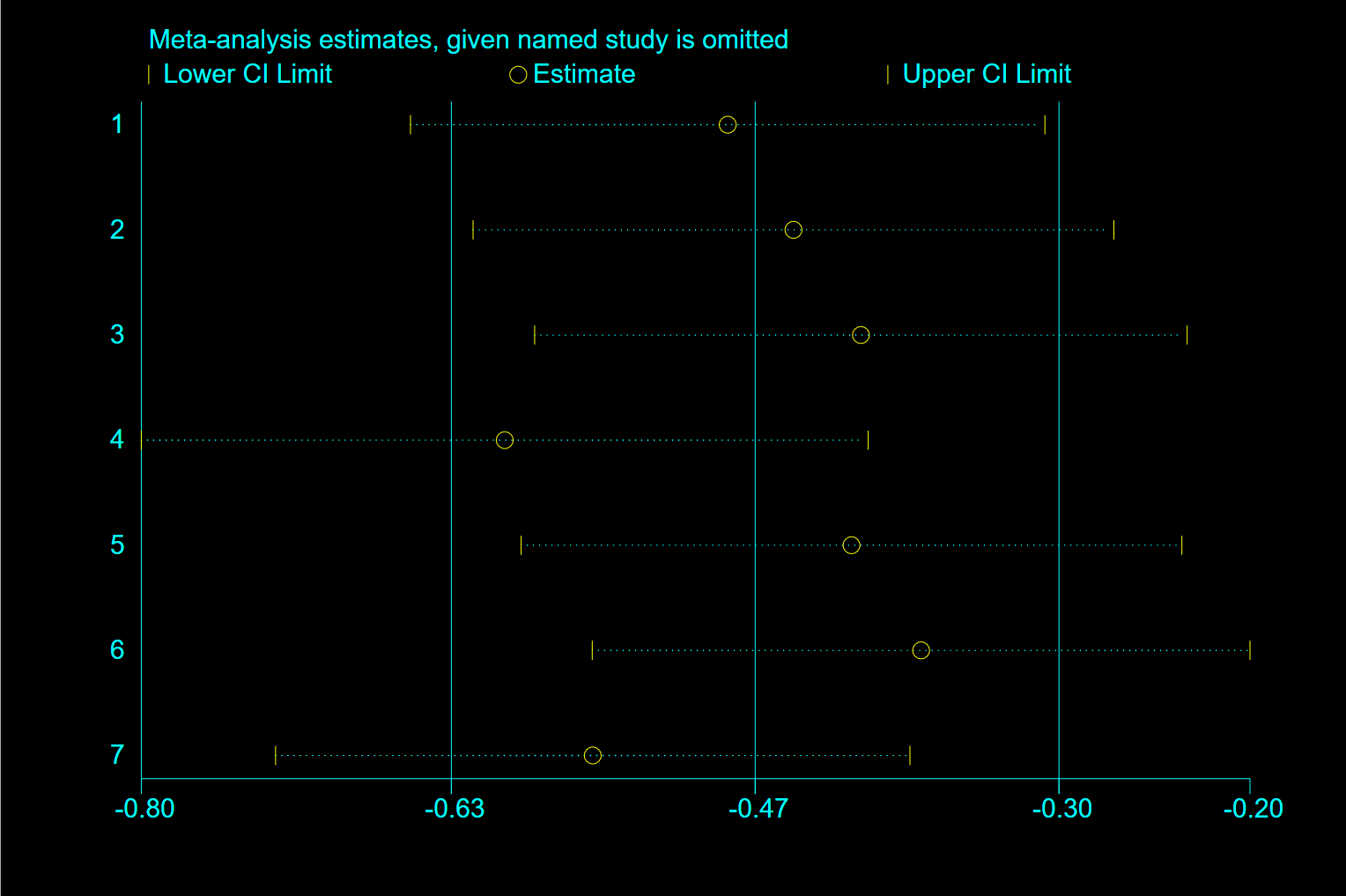
**

**B.
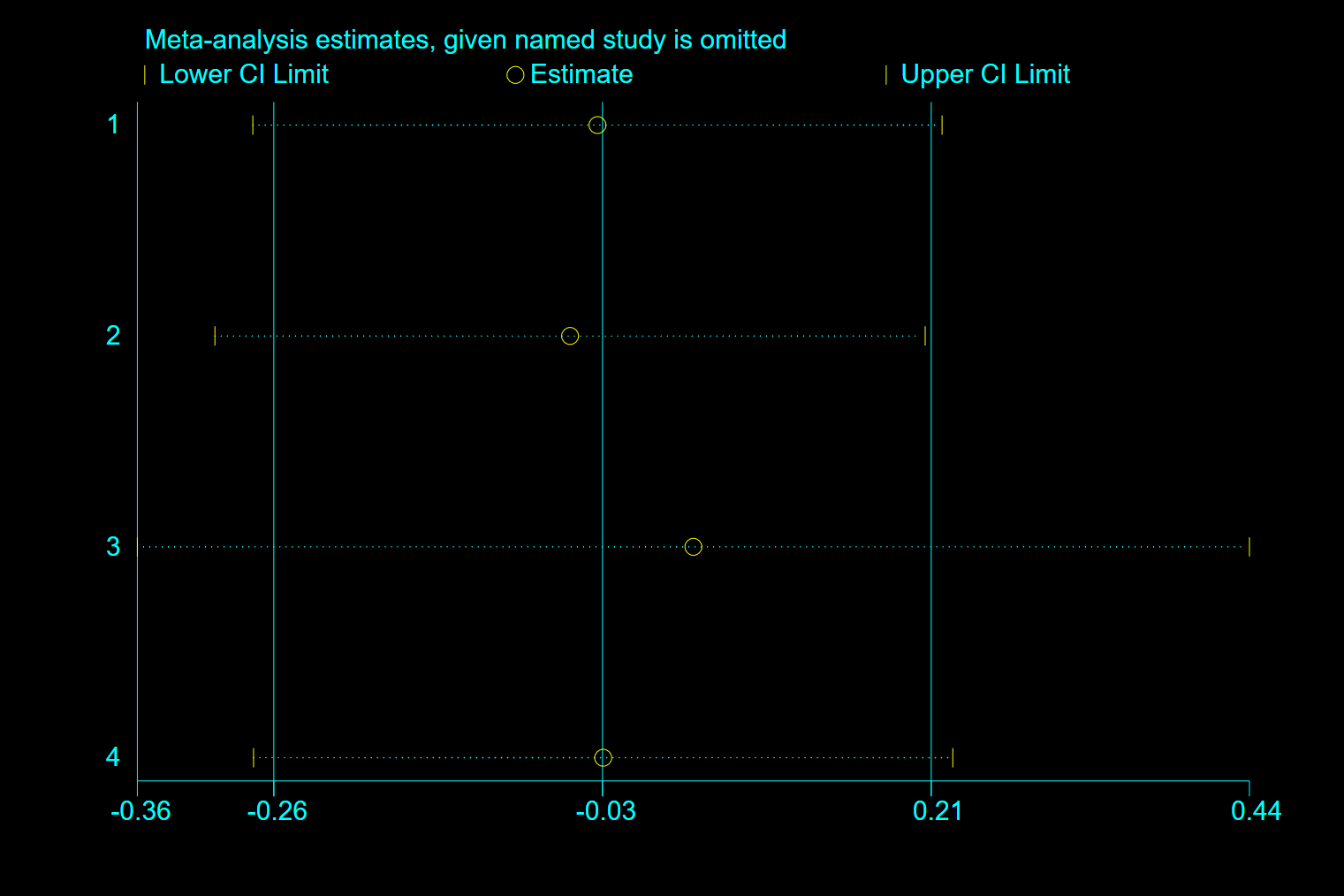
**

**Figure S2 Sensitivity Analysis Plots for PFS and OS. (A) PFS; (B) OS. progression-free survival (PFS); overall survival (OS).**

**A.
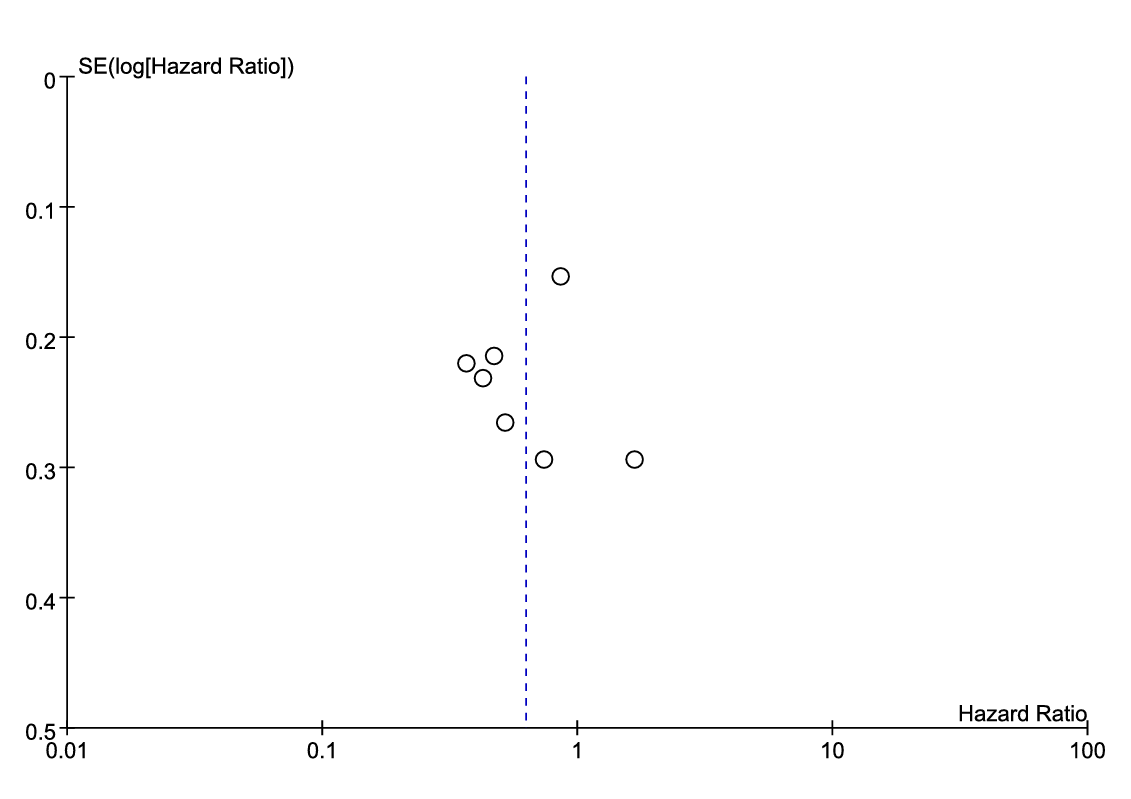
B.
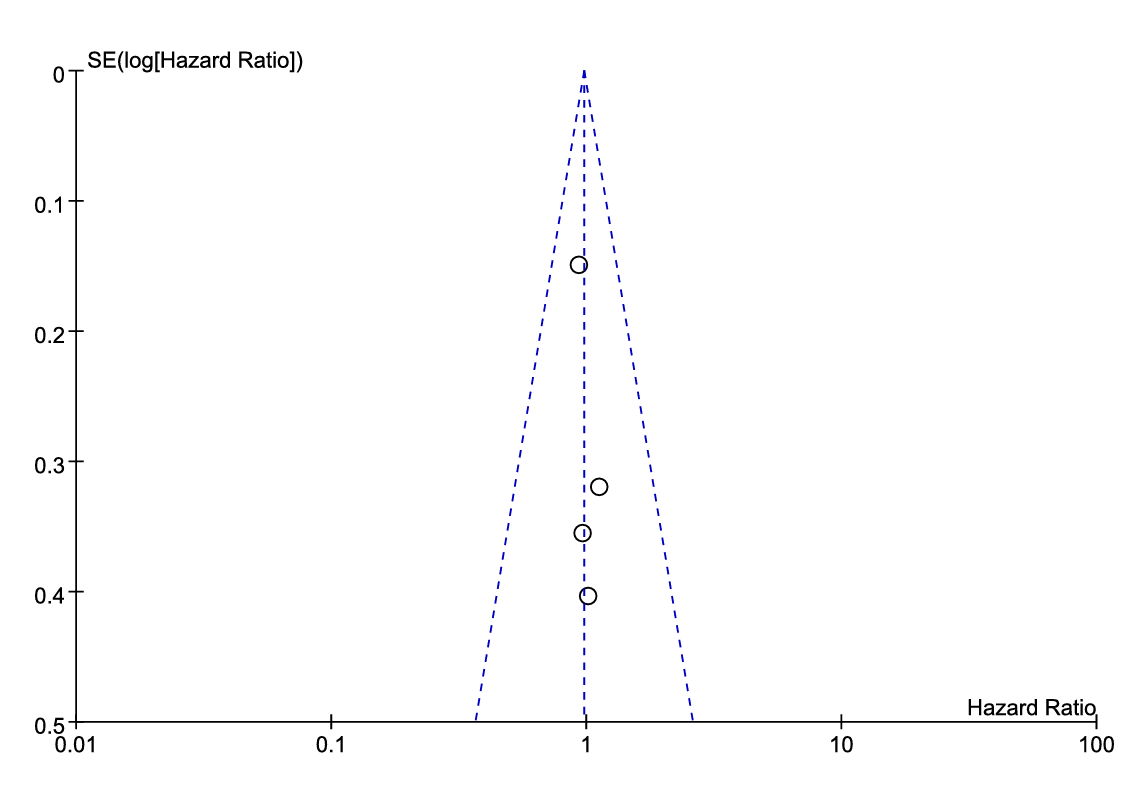
**

**Figure S3 Funnel plot of PFS and OS. (A) PFS; (B) OS. progression-free survival (PFS); overall survival (OS).**

**A.
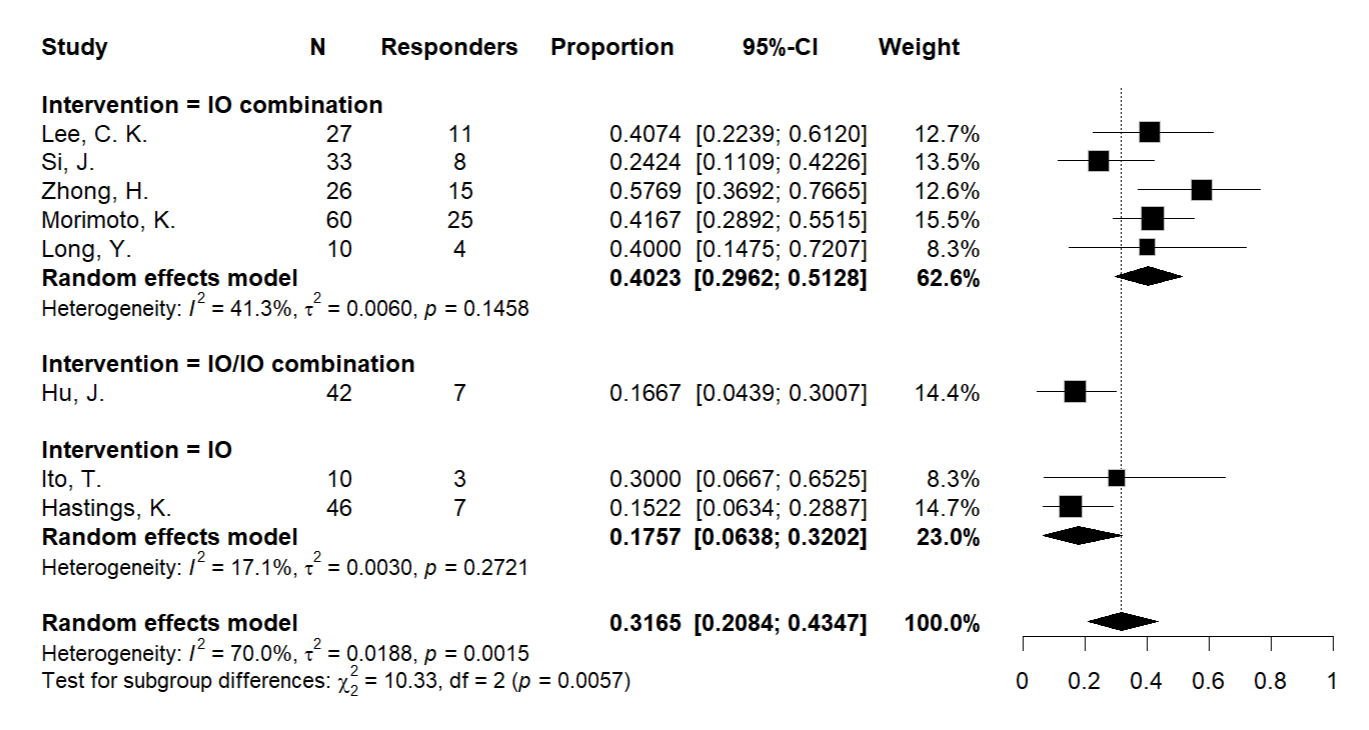
**

**B.
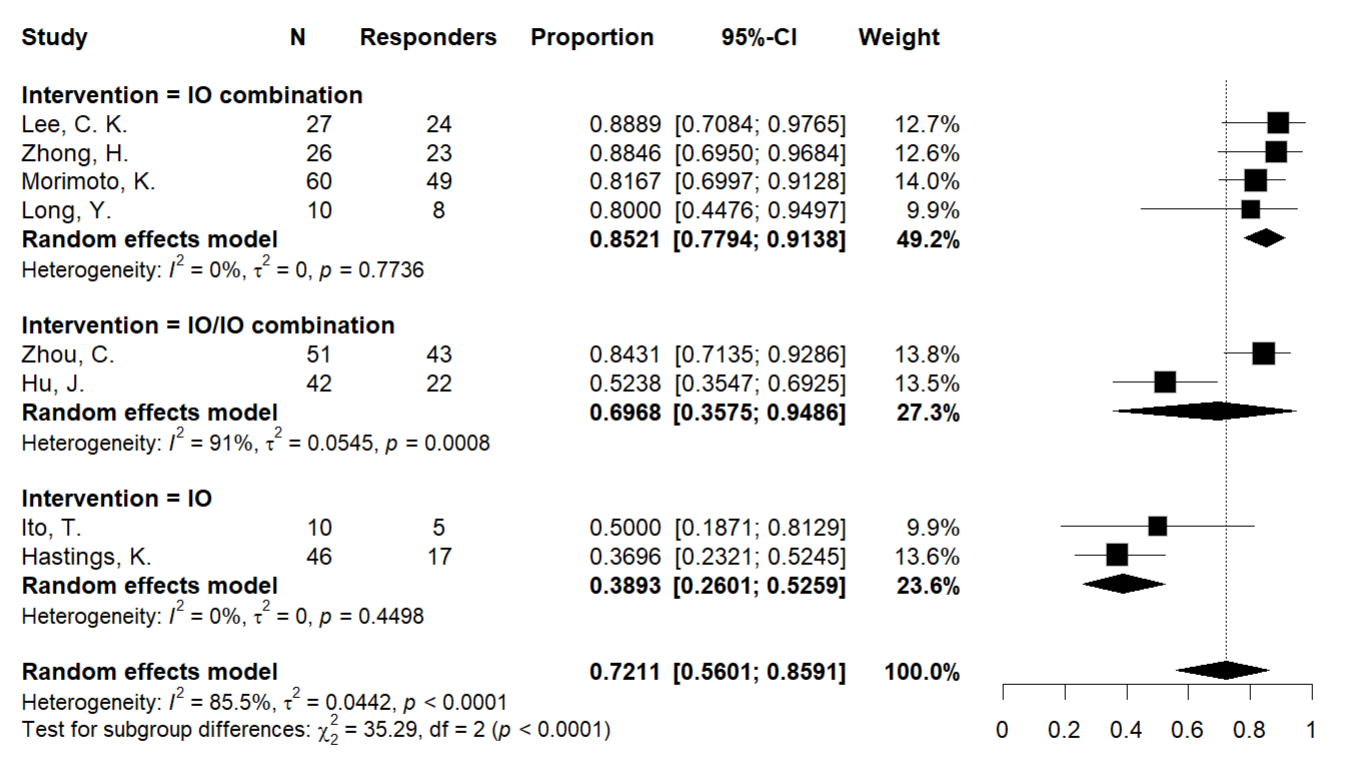
**

**Figure S4 Forest plot of subgroup analysis based on different treatment approaches. (A) ORR; (B) DCR.** **overall response rate (ORR); disease control rate (DCR).**

**A.
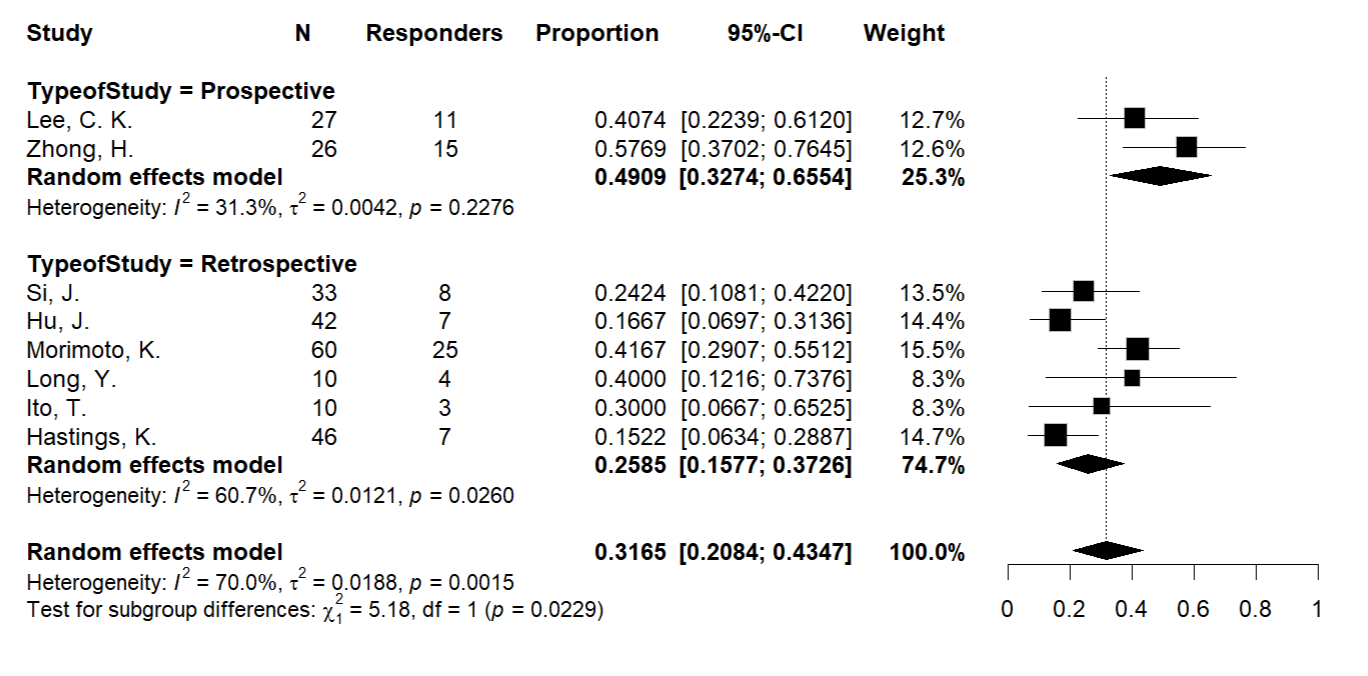
**

**B.
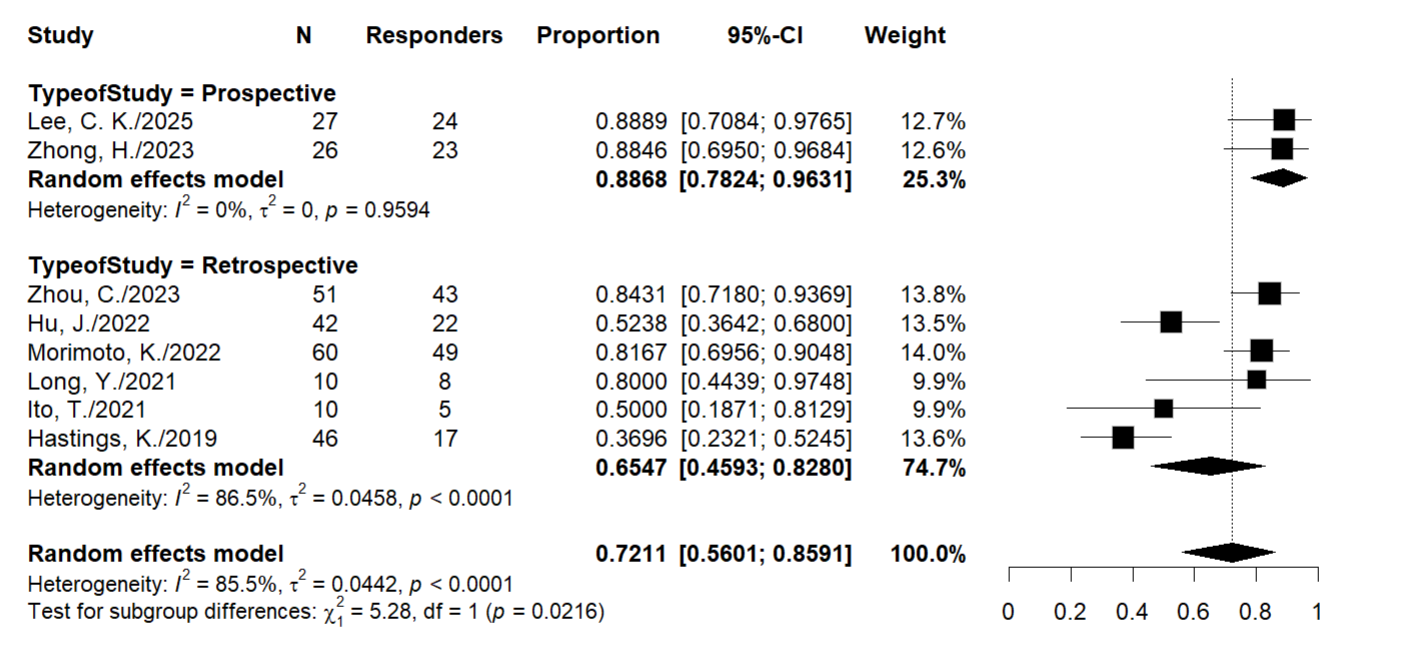
**

**Figure S5 Forest plot of subgroup analysis based on different study types. (A) ORR; (B) DCR.** **overall response rate (ORR); disease control rate (DCR).**

**A.
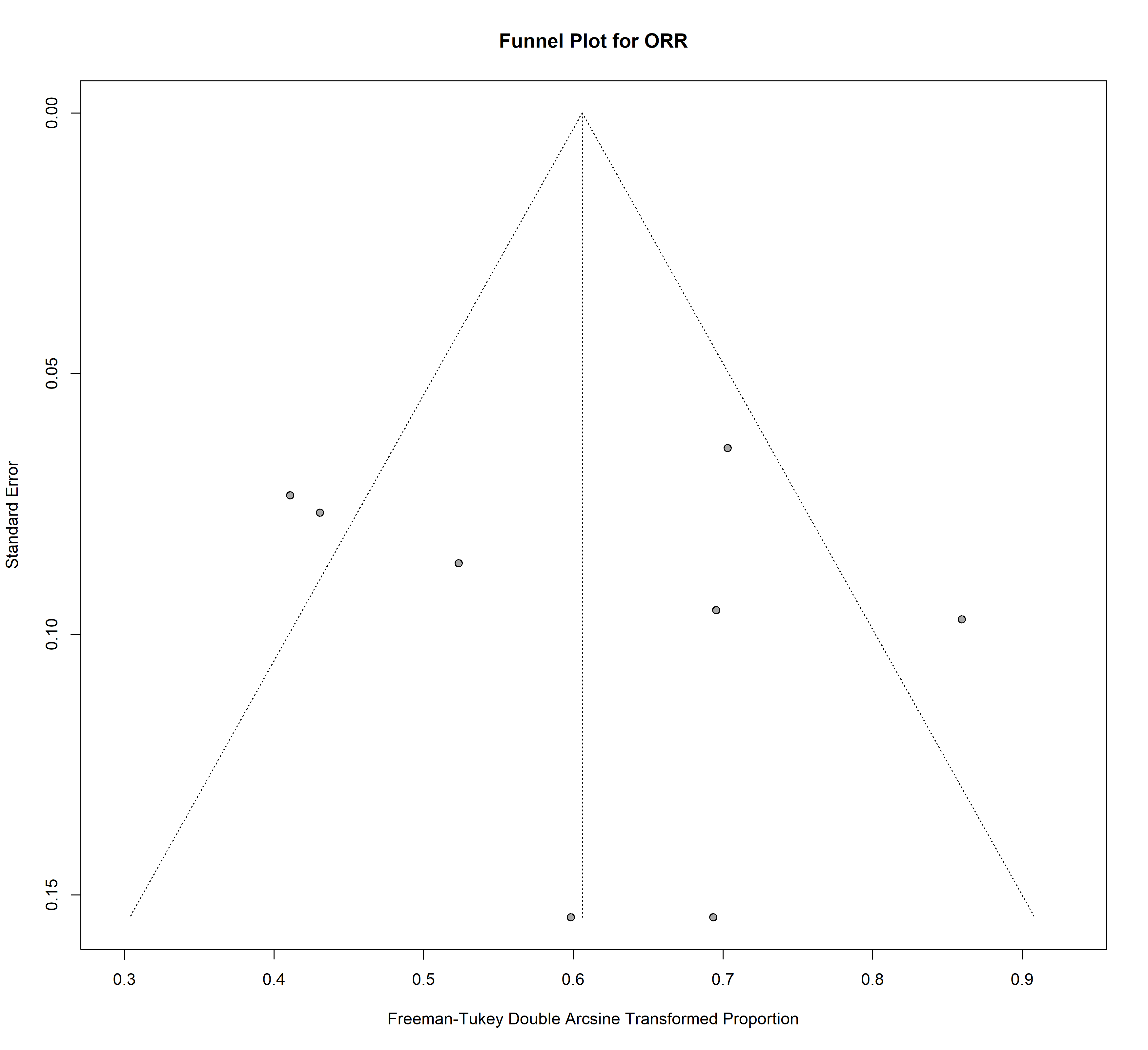
B.
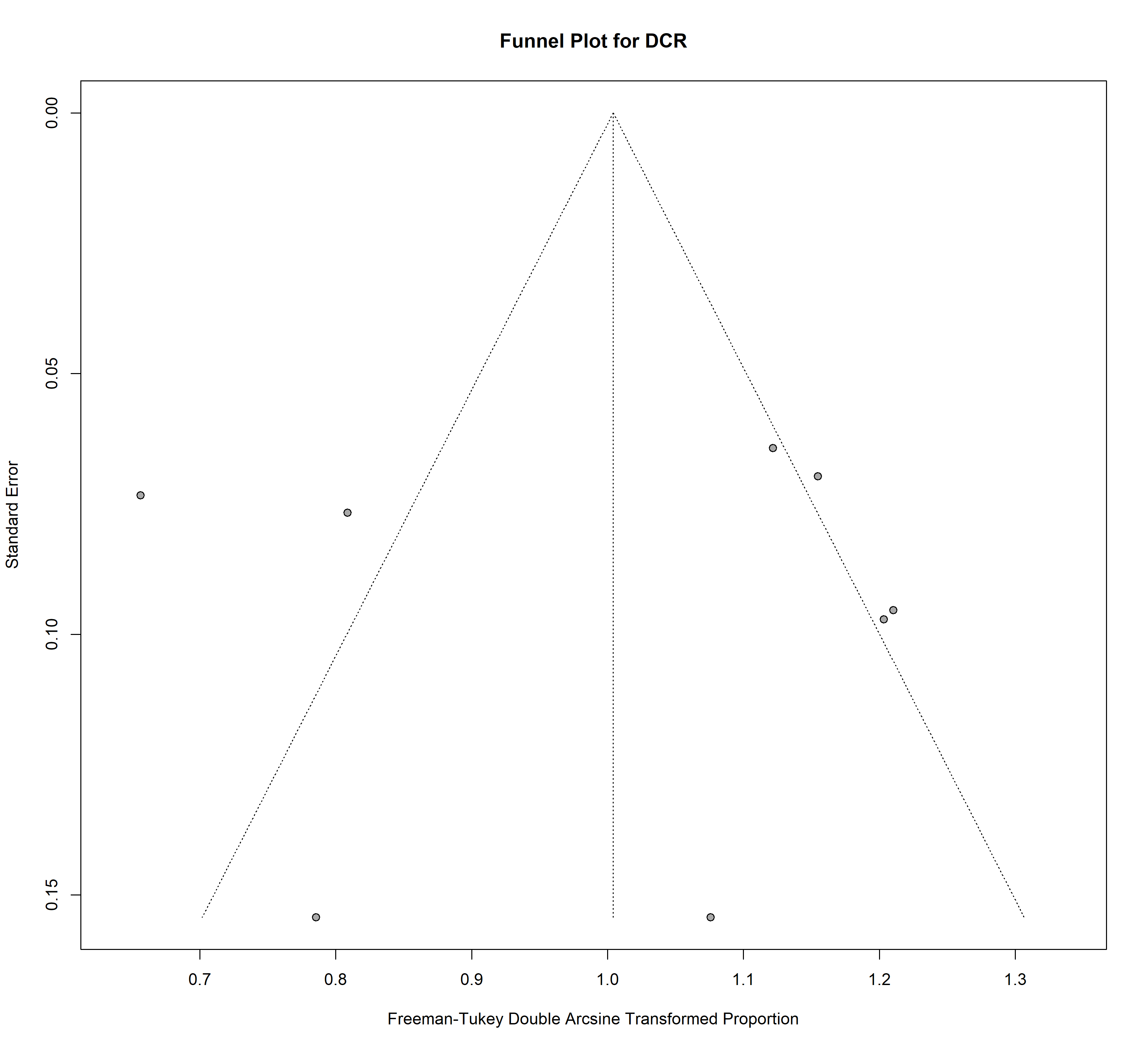
**

**Figure S6 Funnel plot of single-arm ORR and DCR.** **(A) ORR; (B) DCR.** **overall response rate (ORR); disease control rate (DCR).**

**A.
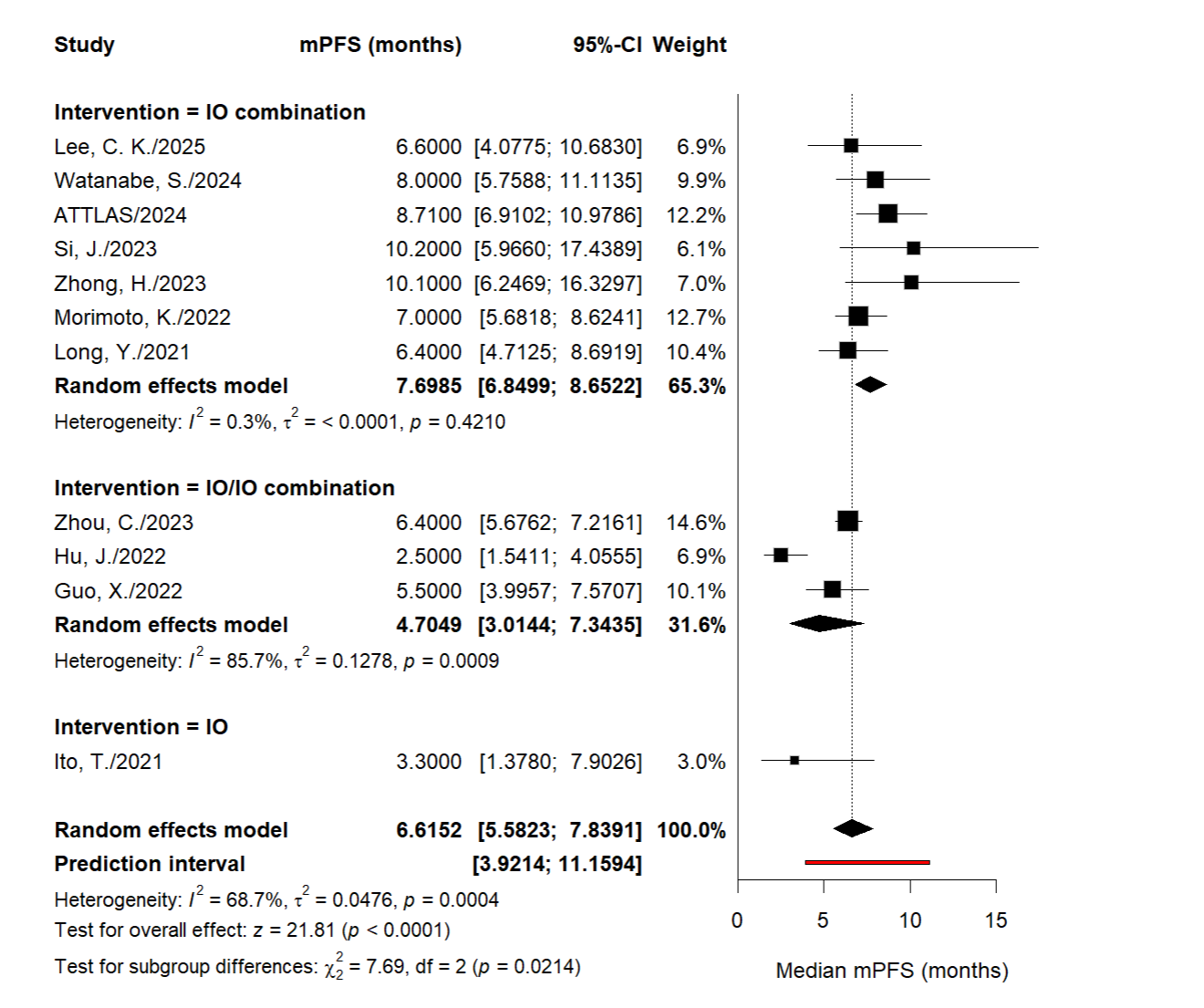
B.
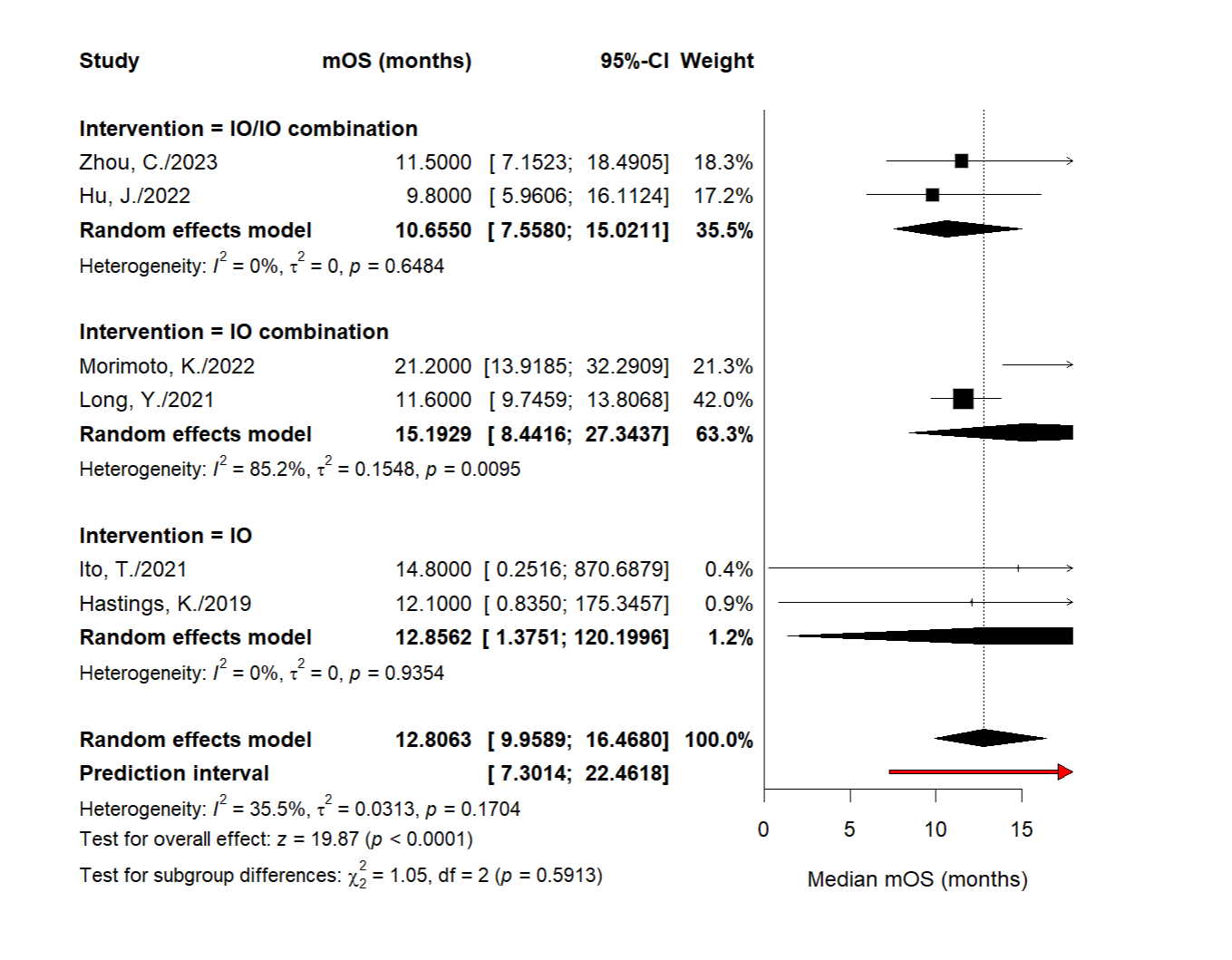
**

**Figure S7 Forest plot of subgroup analysis based on different treatment approaches. (A) PFS; (B) OS.** **progression-free survival (PFS); overall survival (OS).**

**
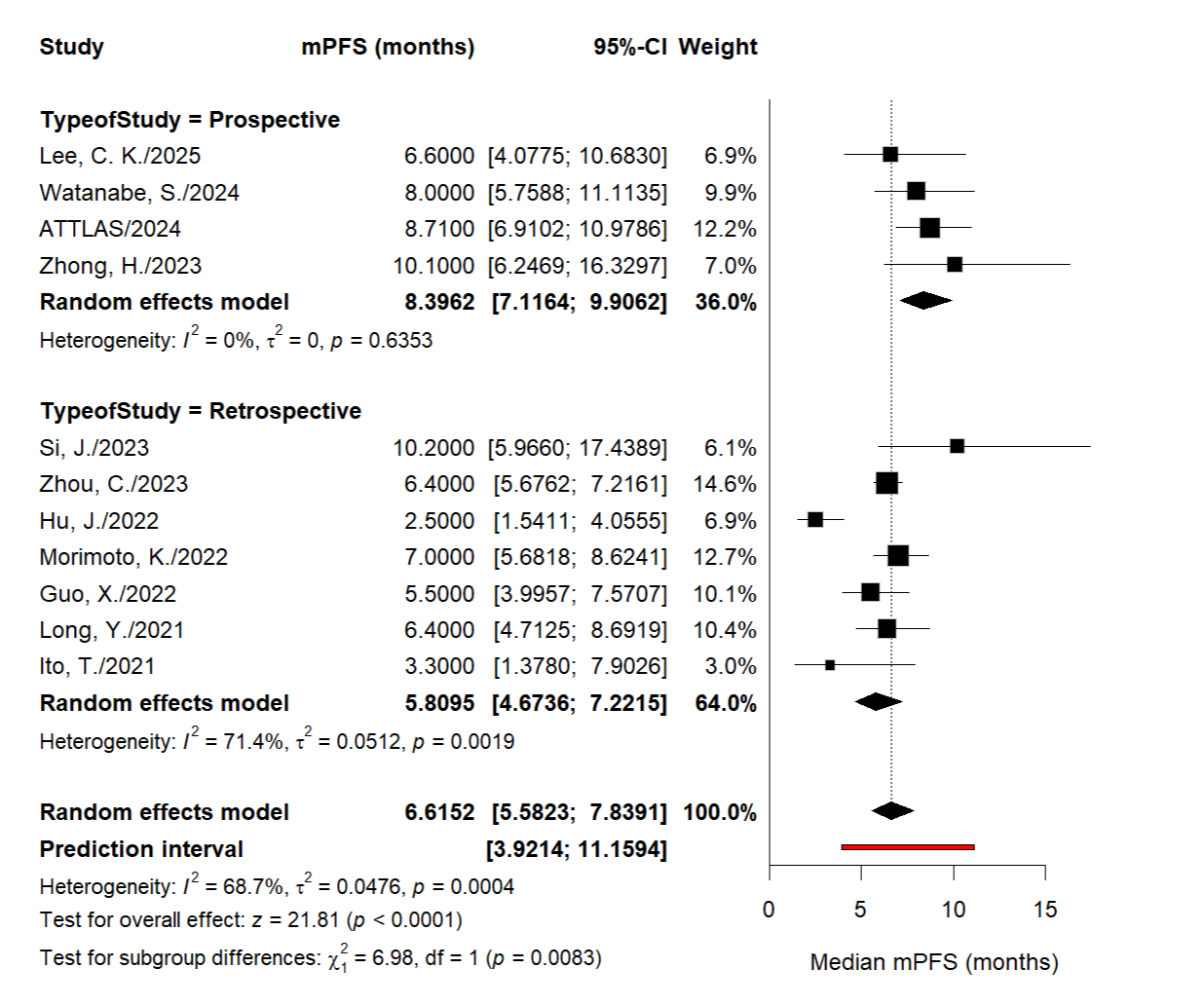
**

**Figure S8 Forest plot of single-arm subgroup analysis of PFS based on different study types.**

**A.
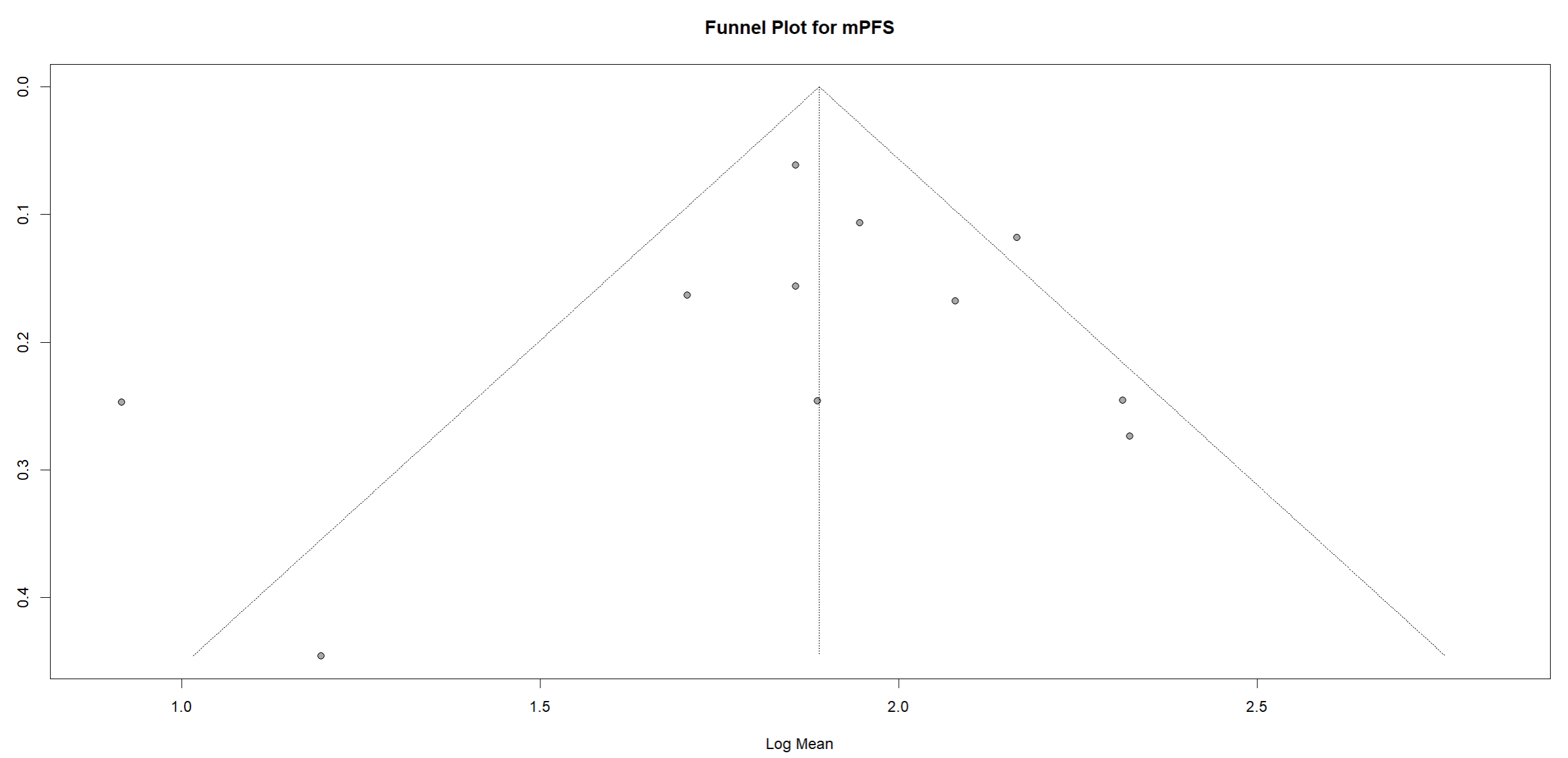
**

**B.
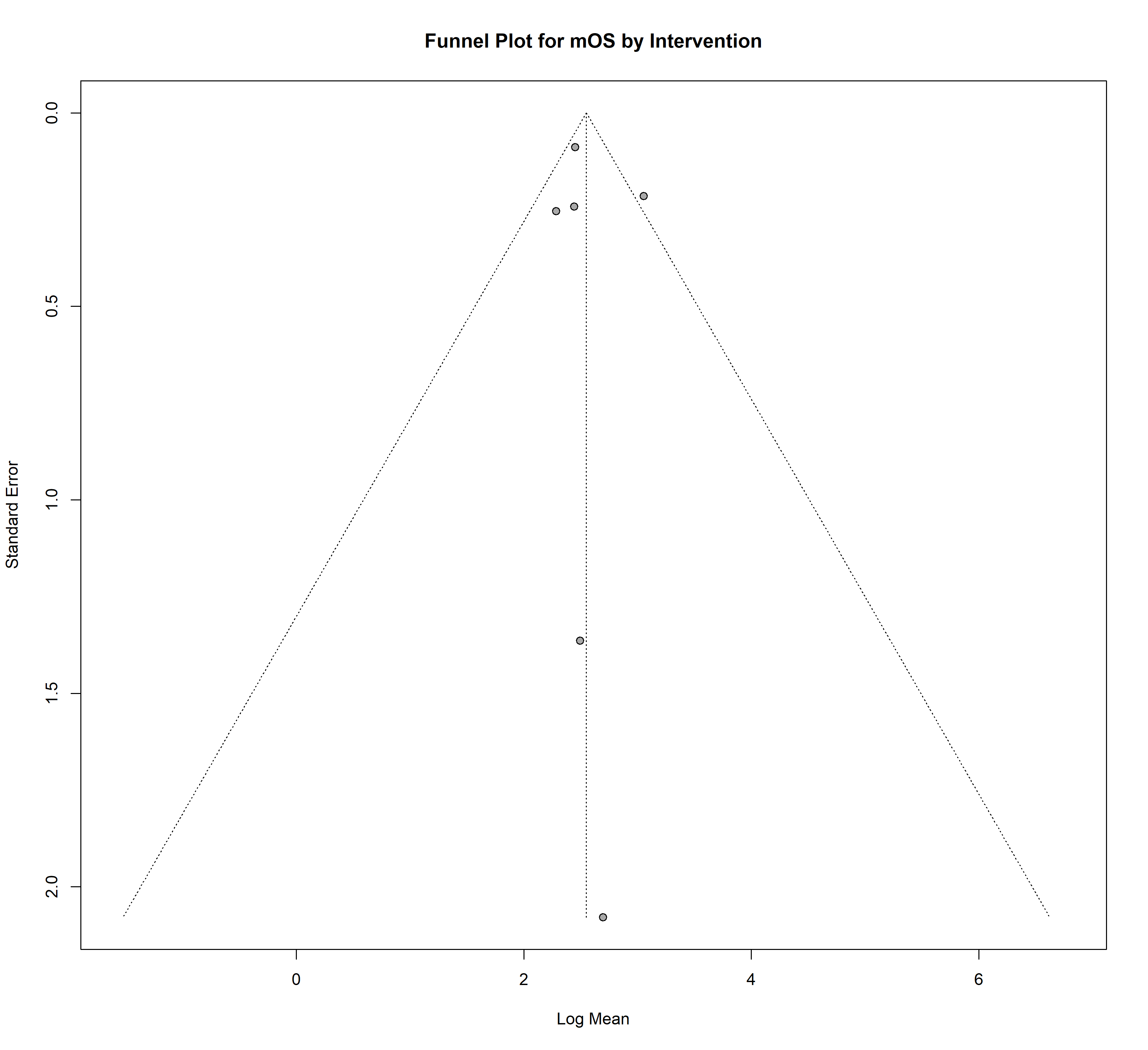
**

**Figure S9 Funnel plot of single-arm PFS and OS studies. (A) PFS; (B) OS.** **progression-free survival (PFS); overall survival (OS).**
