# Supplementary material for: Immunotherapy for TKI-resistant, EGFR L858R-mutated non-small cell lung cancer: a systematic review and meta-analysis of randomized and single-arm studies
Source: Front Immunol. 2026 Apr 10;17:1787310. doi: 10.3389/fimmu.2026.1787310 (PMC13106209; doi:10.3389/fimmu.2026.1787310)
Supplement: Supplementary file 1 [file DataSheet1.zip › Supplementary Material Presentation/Table.Characteristics of the clinical trials included in this study..docx]

**Table 1.** Baseline characteristics of the included randomized controlled trial studies..

| Study | Year | Auther | Phase | Histology | Intervention | Control treatment | OS  HR (95%CI) | PFS  HR (95%CI) |
| --- | --- | --- | --- | --- | --- | --- | --- | --- |
| IMpower151 | 2025 | Zhou, C. | Ⅲ | nsqNSCLC | Atezolizumab+Bev+CT (26) | Bev+CT(31) | 1.01 (0.46, 2.23) | 0.74(0.42-1.33) |
| ATTLAS | 2024 | Park, S. | Ⅲ | nsqNSCLC | Atezolizumab+ Bev +CT(72) | CT(25) | 1.12(0.60-2.10) | 0.52(0.31-0.88) |
| HARMONi-A | 2024 | Fang, W. | Ⅲ | nsqNSCLC | Ivonescimab+CT (60) | CT(78) | - | 0.43(0.27-0.67) |
| KEYNOTE-789 | 2024 | Yang, J. C. H. | Ⅲ | nsqNSCLC | pembrolizumab+CT (103) | CT(102) | 0.94(0.70-1.26) | 0.86(0.64-1.17) |
| ORIENT-31 | 2023 | Lu, S. | Ⅱ | nsqNSCLC | Sintilimab+CT (62) | CT(61) | - | 0·47 (0·31–0·72) |
|  |  |  |  | nsqNSCLC | sintilimab+IBI305+CT (70) | CT(61) |  | 0.37(0.24-0.57) |
| WJOG8515L | 2021 | Hayashi, H. | Ⅱ | nsqNSCLC | Nivolumab  (28) | CT(24) | 0.97(0.48-1.93) | 1.68(0.94-2.98) |

Abbreviations: CT, chemotherapy; nsq, non-squamous; PFS, progression-free survival; OS, overall survival; HR, hazard ratio; CI, confidence interval; Bev, Bevacizumab.

**Table 2.** Baseline characteristics of the included non-randomized controlled trial studies.

| Auther | Year | TypeofStudy | Histology | Intervention | ORR^a^, % (95%CI) | DCR^c^, % (95%CI) | mOS (95%CI),  mo | mPFS (95%CI), mo |
| --- | --- | --- | --- | --- | --- | --- | --- | --- |
| Lee, C. K. | 2025 | Prospective | nsqNSCLC | IO combination(27) | 41.0(25.0-59.0) | 88.9(72.6-96.9） | - | 6.6(5.0–13.1) |
| Watanabe, S. | 2024 | Prospective | nsqNSCLC | IO combination(17) | - | - | - | 8.0(5.7-11.0) |
| ATTLAS | 2024 | Prospective | nsqNSCLC | IO combination(72) | - | - | - | 8.71(6.93-11.01) |
| Si, J. | 2023 | Retrospective | nsqNSCLC | IO combination(33) | 24.2(11.1-42.3) | - | - | 10.2(5.2-15.2) |
| Zhou, C. | 2023 | Retrospective | nsqNSCLC | IO/IO combination(51) | - | 84.3 | 11.5(5.69–14.71) | 6.4(5.64–7.17) |
| Zhong, H. | 2023 | Prospective | nsqNSCLC | IO combination(26) | 57.7(38.4-75.1) | 88.5(71.9-96.8) | - | 10.1(5.7-14.9) |
| Hu, J. | 2022 | Retrospective | NSCLC | IO/IO combination(42) | 16.7 | 52.4(36.9-67.5) | 9.8(6.4-17.3) | 2.5(1.9-5.0) |
| Morimoto, K. | 2022 | Retrospective | NSCLC | IO combination (60) | 42.4(29.8-55.8) | 81.8(70.1-90.2) | 21.2(15.0-34.8) | 7.0(5.6-8.5) |
| Guo, X. | 2022 | Retrospective | nsqNSCLC | IO/IO combination(29) |  | - | - | 5.5(3.8-7.2) |
| Long, Y. | 2021 | Retrospective | NSCLC | IO combination (10) | 40.0(12.2-73.8) | 80.0(44.4-97.5) | 11.6(9.6–13.6) | 6.4(4.5–8.3) |
| Ito, T. | 2021 | Retrospective | NSCLC | IO(10) | 30.0 | 50.0 | 14.8(0.00–34.61) | 3.3(0.98-5.62) |
| Hastings, K. | 2019 | Retrospective | NSCLC | IO(46) | 15.2(6.7-27.4) | 36.9(23.2-52.2) | 12.1(0.3–63) | - |

Abbreviations: nsq, non-squamous; PFS, progression-free survival; OS, overall survival; CI, confidence interval; ORR, overall response rate; DCR ,disease control rate; IO, Immunotherapy.
