# Supplementary material for: Immunotherapy for TKI-resistant, EGFR L858R-mutated non-small cell lung cancer: a systematic review and meta-analysis of randomized and single-arm studies
Source: Front Immunol. 2026 Apr 10;17:1787310. doi: 10.3389/fimmu.2026.1787310 (PMC13106209; doi:10.3389/fimmu.2026.1787310)
Supplement: Supplementary file 1 [file DataSheet1.zip › Supplementary Material Presentation/Title Page.docx]

**Immunotherapy for TKI-Resistant, EGFR L858R-Mutated Non-Small Cell Lung Cancer: A Systematic Review and Meta-Analysis of Randomized and Single-Arm Studies**

Peipei Zhang*^1^**, Weixing Zhao*^1^**, Jiayun Ma*^1^** ,Yuan Li*^1^* , Huiyuan Peng*^1^* , Jun Jiang*^2^*#

*^1^Department of Oncology, Graduate School of Qinghai University, Qinghai, China, ^2^Division III, Department of Medical Oncology, Qinghai University Affiliated Hospital, Qinghai, China*

* These authors contributed equally to this work

**Correspondence:** Jun Jiang MD, PhD, *Department of Medical Oncology, Qinghai University Affiliated Hospital,* 29, Tongren Road Chengxi District, Xining City Qinghai Province, China. Tel: 18997268135; Fax: 18997268135, E-mail: [xnrheum@126.com](mailto:xnrheum@126.com) ORCID: <https://orcid.org/0000-0002-6248-6179>

**Acknowledgments:** The article uses the AI large model (Gemini 3.0) for language translation and refinement.

**Authorship contributions:**

**Peipei Zhang:** Conceptualization; Formal analysis; Methodology; Validation; Writing – original draft; Writing – review & editing;

**Weixing Zhao:**Conceptualization; Methodology; Software; Visualization; Writing – original draft; Writing – review & editing;

**Jiayun Ma:**  Conceptualization; Formal analysis; Resources; Supervision; Writing – original draft; Writing – review & editing;

**Yuan Li:** Software; Supervision; Writing – original draft; Writing – review & editing;

**Huiyuan Peng:** Formal analysis; Investigation; Writing – original draft; Writing – review & editing;

**Jun Jiang:** Conceptualization; Funding acquisition; Methodology; Writing – original draft; Writing – review & editing.

**Statements and declarations**

**Ethics approval and consent to participate Not applicable.**

**Funding:**The author(s) disclosed receipt of the following financial support for the research, authorship, and/or publication of this article: This paper is funded by the Science and Technology Support Qinghai Project（2025-QY-253）from Science and Technology Department of Qinghai Province.

**Conflict of interests**: The authors declare that they have no competing fnancial interests or personal relationships that may have infuenced the work reported in this study.

**Consent for publication:** Not applicable.

**Data availability：**All data generated or analysed during this study are included in this article. Further enquiries can be directed to the corresponding author.
