# Supplementary figures and images for: Immunotherapy for TKI-resistant, EGFR L858R-mutated non-small cell lung cancer: a systematic review and meta-analysis of randomized and single-arm studies
Source: Front Immunol. 2026 Apr 10;17:1787310. doi: 10.3389/fimmu.2026.1787310 (PMC13106209; doi:10.3389/fimmu.2026.1787310)

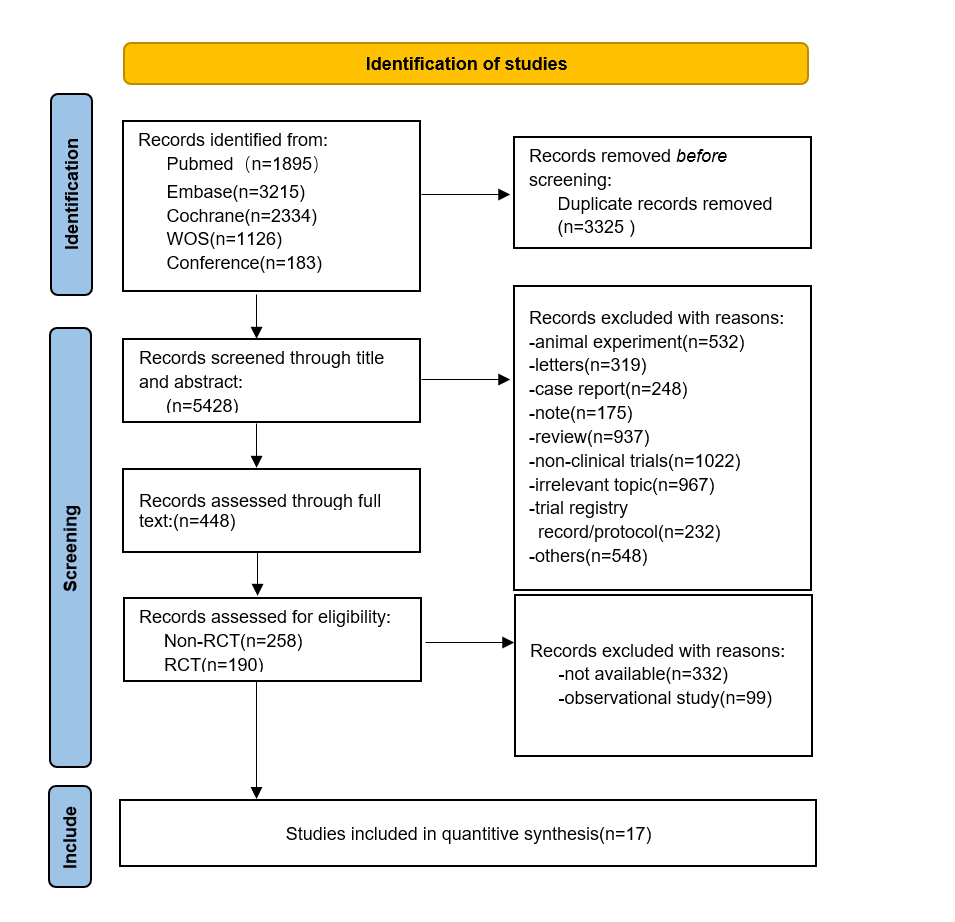

Supplement: Supplementary file 1 [file DataSheet1.zip › Supplementary Material Presentation/Figures/Figure 1.png]

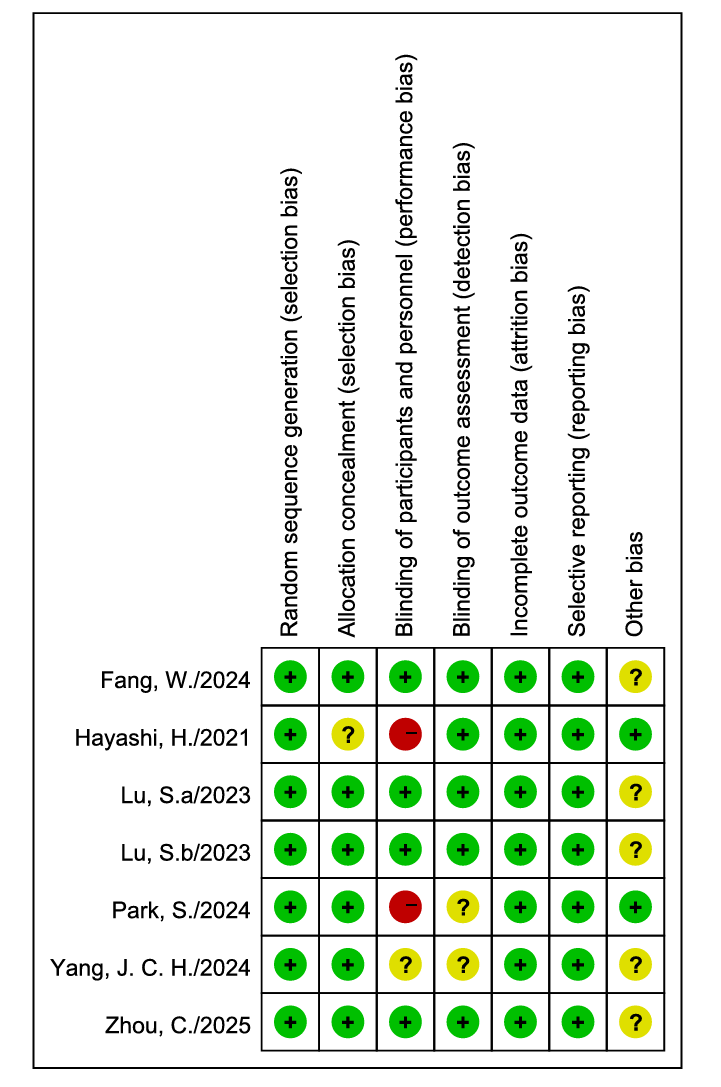

Supplement: Supplementary file 1 [file DataSheet1.zip › Supplementary Material Presentation/Figures/Figure 2(A).png]

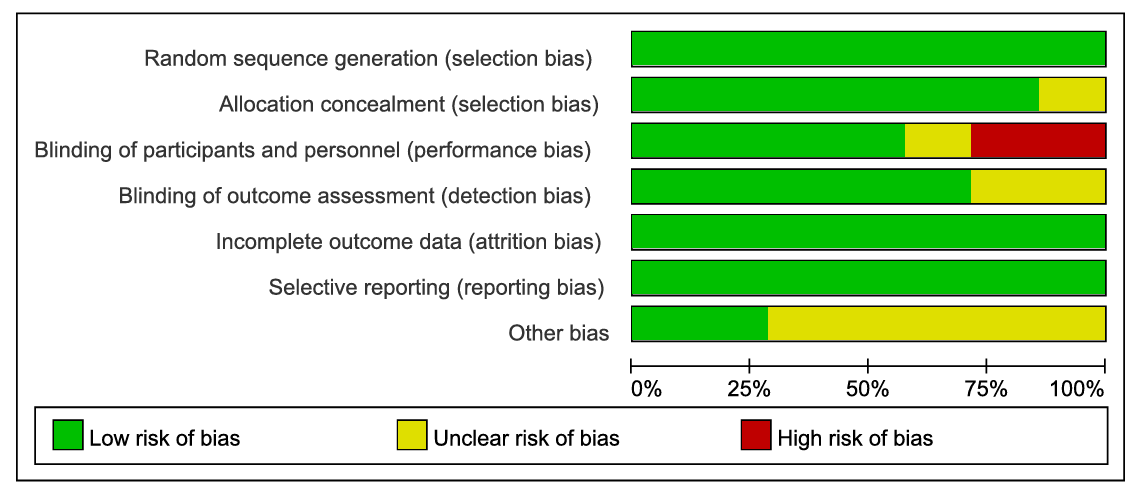

Supplement: Supplementary file 1 [file DataSheet1.zip › Supplementary Material Presentation/Figures/Figure 2(B).png]

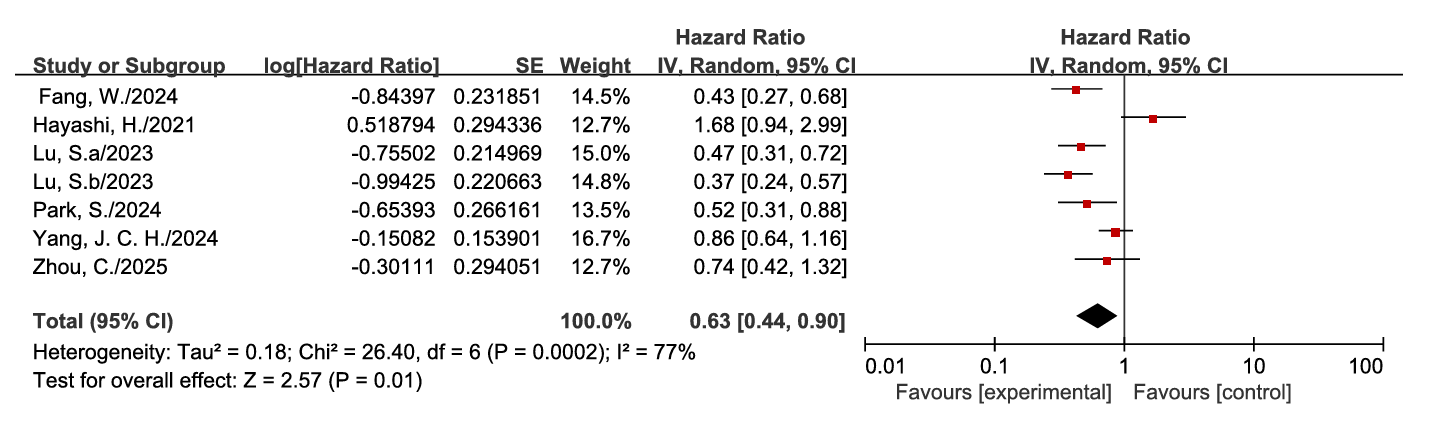

Supplement: Supplementary file 1 [file DataSheet1.zip › Supplementary Material Presentation/Figures/Figure 3(A).png]

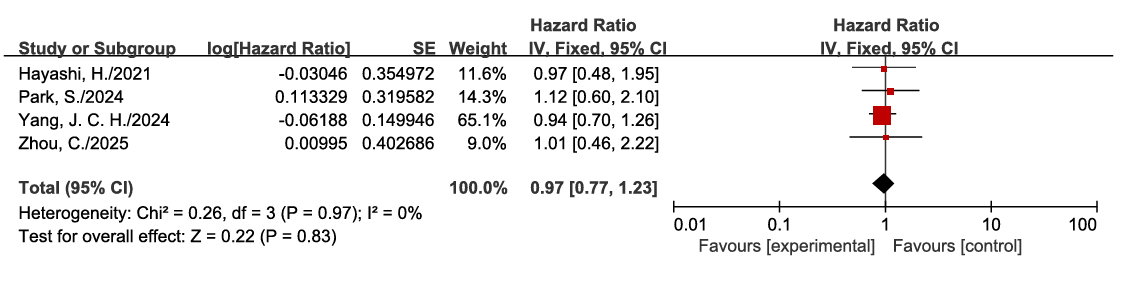

Supplement: Supplementary file 1 [file DataSheet1.zip › Supplementary Material Presentation/Figures/Figure 3(B).png]

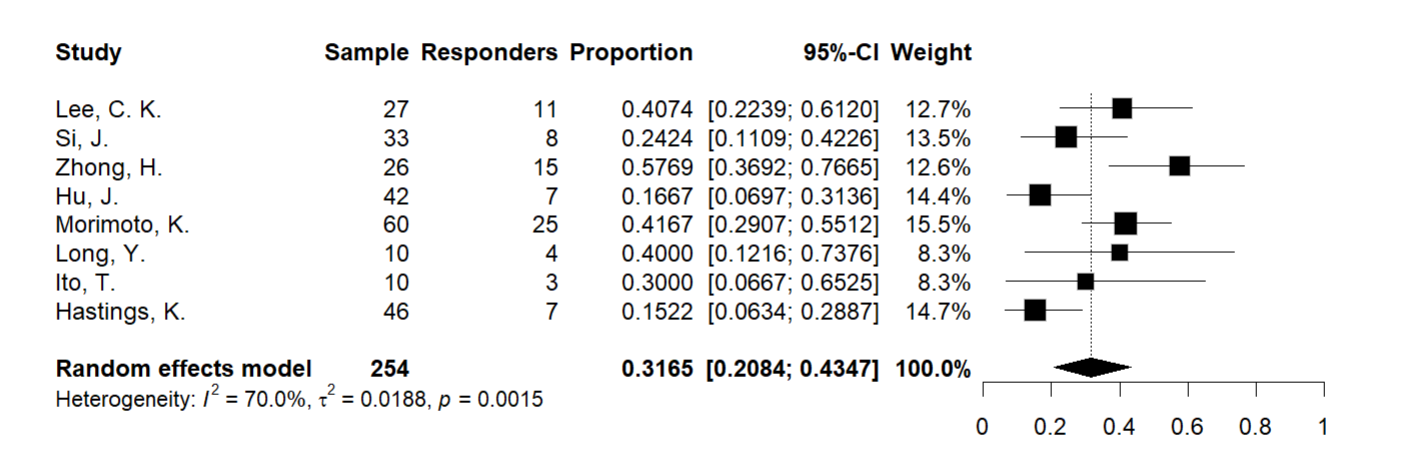

Supplement: Supplementary file 1 [file DataSheet1.zip › Supplementary Material Presentation/Figures/Figure 4(A).png]

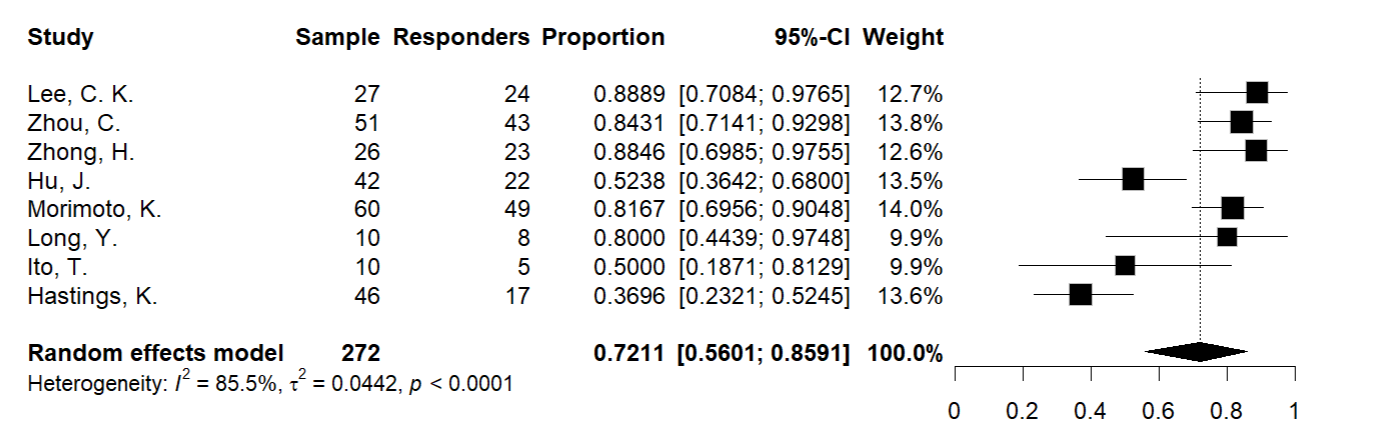

Supplement: Supplementary file 1 [file DataSheet1.zip › Supplementary Material Presentation/Figures/Figure 4(B).png]

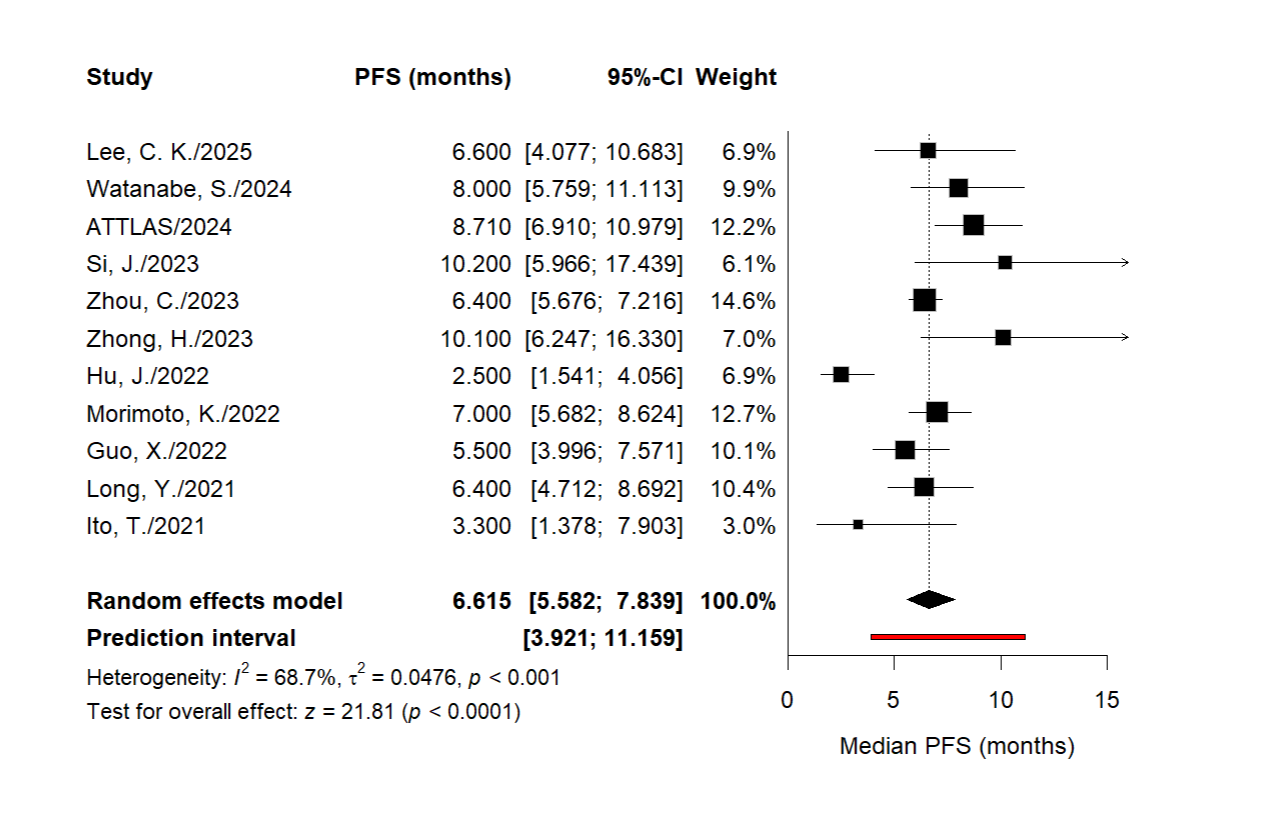

Supplement: Supplementary file 1 [file DataSheet1.zip › Supplementary Material Presentation/Figures/Figure 5(A).png]

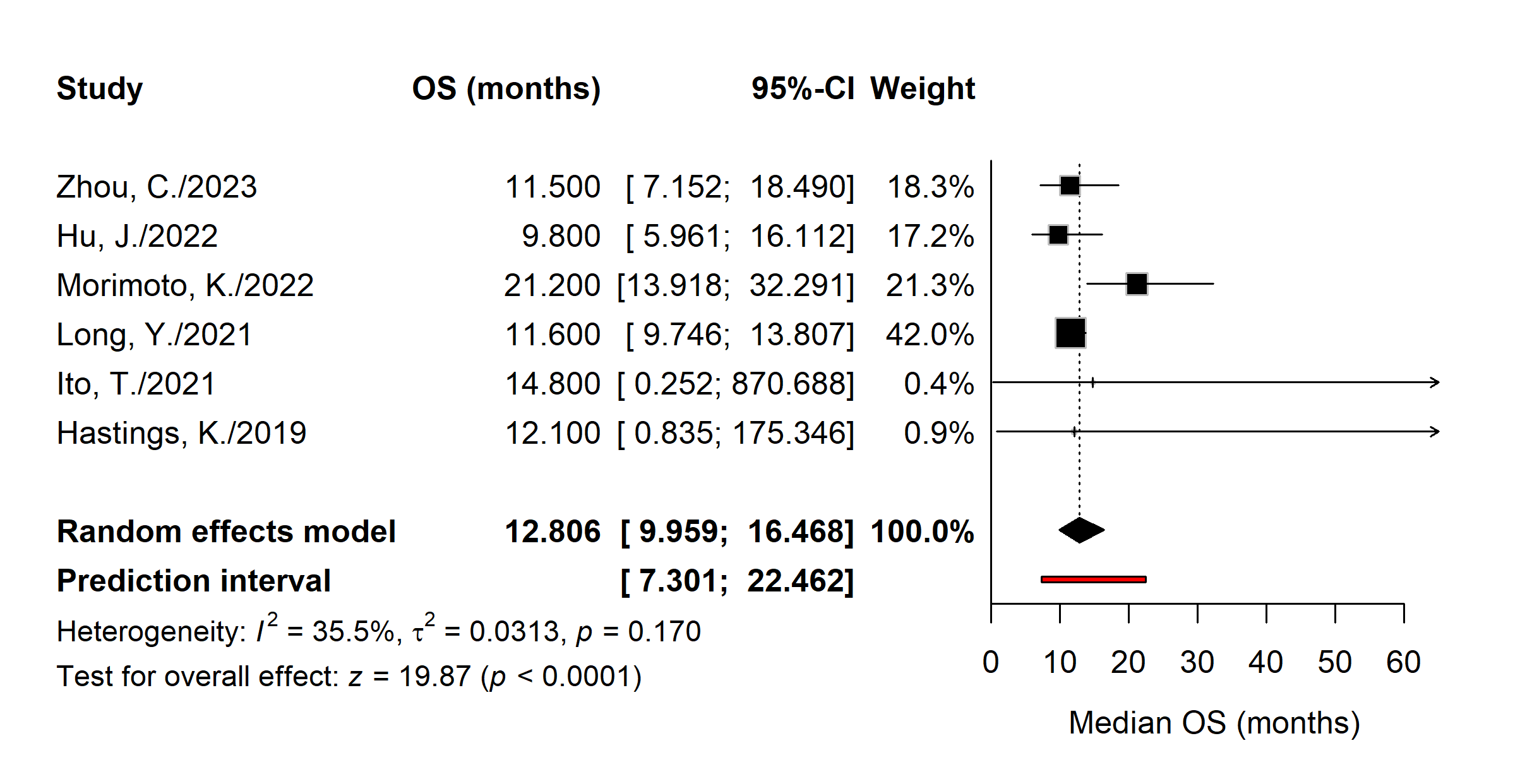

Supplement: Supplementary file 1 [file DataSheet1.zip › Supplementary Material Presentation/Figures/Figure 5(B).png]
