# Supplementary material for: Immunotherapy for TKI-resistant, EGFR L858R-mutated non-small cell lung cancer: a systematic review and meta-analysis of randomized and single-arm studies
Source: Front Immunol. 2026 Apr 10;17:1787310. doi: 10.3389/fimmu.2026.1787310 (PMC13106209; doi:10.3389/fimmu.2026.1787310)
Supplement: Supplementary file 1 [file DataSheet1.zip › Supplementary Material Presentation/Cover letter.docx]

From: Jun Jiang MD, PhD, *Department of Medical Oncology, Qinghai University Affiliated Hospital,* 29, Tongren Road Chengxi District, Xining City Qinghai Province, China. Tel: 18997268135; Fax: 18997268135, E-mail: xnrheum@126.com

# March 17, 2026

To: Editor-in-Chief

[Frontiers in Immunology](https://www.frontiersin.org/journals/immunology)

Dear Editor**:**

**Re: Submission of Manuscript**

We are pleased to submit our original research article entitled “Immunotherapy for TKI-Resistant, EGFR L858R-Mutated Non-Small Cell Lung Cancer: A Systematic Review and Meta-Analysis of Randomized and Single-Arm Studies” for consideration for publication in *frontiers in immunology*.The application of immune checkpoint inhibitors (ICIs) in *EGFR*-mutated non-small cell lung cancer (NSCLC) following tyrosine kinase inhibitor (TKI) resistance remains a subject of intense debate. While previous meta-analyses have attempted to evaluate ICI efficacy in this setting, they have predominantly analyzed exon 19 deletions and L858R mutations in aggregate. This approach obscures the significant immunobiological heterogeneity between these subtypes, particularly given evidence that L858R-mutated tumors possess a distinct, generally "colder" tumor microenvironment.

To address this critical knowledge gap, our study represents the first systematic review and meta-analysis to focus exclusively on the *EGFR* L858R subtype. By synthesizing data from 17 prospective and retrospective studies involving 1,154 patients, we provide robust evidence tailored to this specific patient population.

Our key findings include:

1.Inefficacy of Monotherapy: We confirm that ICI monotherapy fails to confer a survival benefit in L858R-mutated patients, supporting the biological hypothesis of intrinsic resistance in this subtype.

2.Superiority of Combination Strategies: We demonstrate that ICI-based combinations significantly delay disease progression. Notably, the “four-drug regimen” (ICI + anti-angiogenic agents + chemotherapy) exhibited the optimal progression-free survival benefit (HR=0.50, P<0.01), outperforming standard chemotherapy and other ICI combinations.

3.Clinical Implications: These results provide an evidence-based framework for precision medicine, suggesting that adding anti-angiogenic agents to chemo-immunotherapy may be the key to overcoming the immunosuppressive microenvironment in L858R-mutated NSCLC.

We declare that this manuscript is original, has not been published before, and is not currently being considered for publication elsewhere. All authors have read and approved the final version of the manuscript. The authors declare no conflicts of interest.

Thank you for your time and consideration of our work.

Dr. Jiang will serve as the corresponding author.

Sincerely Yours,

Jun Jiang
